# Telomere Length and Clear Cell Renal Cell Carcinoma: Unraveling Causal Mechanisms Through Integrative Genetic and Single-Cell Transcriptomic Analysis

**DOI:** 10.1155/mi/3705788

**Published:** 2025-11-27

**Authors:** Kaixiang Zhang, Congjun Huang, Jing Zhou, Kai Fan, Ming Ruan, Youping Xiao

**Affiliations:** Department of Urology, Second Affiliated Hospital of Guizhou University of Traditional Chinese Medicine, Guiyang, Guizhou, China

**Keywords:** clear cell renal cell carcinoma, colocalization, Mendelian randomization, single-cell RNA sequencing, telomere length

## Abstract

**Background:**

Clear cell renal cell carcinoma (ccRCC) presents significant clinical challenges. This study investigates the causal relationship between telomere length (TL) and ccRCC risk using a comprehensive multicohort genetic approach.

**Methods:**

We conducted a rigorous two-sample Mendelian randomization (MR) analysis using independent discovery (UK Biobank, *n* = 472,174) and validation (genome-wide association studies [GWAS] Catalog, *n* = 438,351) cohorts. Multivariable MR (MVMR) adjusted for chronic kidney disease (CKD), hypertension, and smoking. Colocalization analysis and single-cell RNA sequencing (scRNA-seq) were employed to validate genetic associations and explore cellular mechanisms.

**Results:**

In the discovery cohort, inverse variance weighted (IVW) MR analysis revealed a significant positive association between TL and ccRCC risk (odds ratio [OR]: 1.604, 95% confidence interval [CI]: 1.358–1.895, *p* < 0.001). The validation cohort consistently confirmed these findings (OR: 1.470, 95% CI: 1.290–1.674, *p* < 0.001). MVMR analysis using IVW method, adjusting for key risk factors, demonstrated a significant association (OR: 2.072, 95% CI: 1.724–2.491, *p* < 0.001). Colocalization analysis showed strong evidence of shared causal variants (posterior probability > 98% in both discovery and validation sets). scRNA-seq revealed that proximal tubule cells (PTCs) with telomerase-associated genes *NOP10* and *NHP2* exhibited complex senescence dynamics, characterizing distinct cellular communication patterns in the tumor microenvironment.

**Conclusion:**

Our comprehensive multiomics study provides robust evidence of a causal relationship between TL and ccRCC risk. By integrating genetic epidemiology and single-cell transcriptomics, we unveil novel molecular mechanisms underlying ccRCC pathogenesis and identify potential therapeutic targets.

## 1. Introduction

Clear cell renal cell carcinoma (ccRCC) is the most prevalent and aggressive histological subtype of renal cancer [1]. It is morphologically defined by characteristic cytological features, such as clear cytoplasm, which is a consequence of intracellular accumulation of lipids and glycogen [2]. These features demonstrate a pronounced propensity for metastatic dissemination and resistance to conventional radiation and chemotherapeutic strategies. ccRCC originates predominantly from the epithelial lining of the proximal convoluted tubule and evolves through a complex interplay of genetic, environmental, and metabolic influences [3]. Established risk factors include tobacco use, hypertension, obesity, and chronic kidney impairment, alongside recurrent somatic mutations in key tumor suppressor genes, such as *VHL*, *PBRM1*, and *BAP1*, which collectively contribute to disease initiation and progression [4, 5]. Despite the emergence of molecularly targeted agents and immune checkpoint inhibitors that have modestly improved outcomes, the 5-year survival rate for patients with advanced or metastatic ccRCC remains unfavorable [6, 7]. This underscores an urgent need to decipher the fundamental molecular mechanisms underlying ccRCC oncogenesis and progression.

Telomeres, comprising repetitive nucleotide sequences and an associated protein complex situated at the termini of eukaryotic chromosomes, are indispensable for preserving chromosomal stability and integrity [8]. They prevent end-to-end fusion and degradation, thereby safeguarding the genome [9]. Telomere length (TL) is maintained through a balance of progressive attrition and elongation mechanisms. The attrition occurs due to the end-replication problem and oxidative damage, while the elongation is primarily mediated by the enzyme complex telomerase [10, 11]. Critically shortened telomeres trigger replicative senescence or apoptosis, which serves as a barrier to malignant transformation [12, 13]. Conversely, aberrant telomere maintenance, often through reactivation of telomerase, is a hallmark of cancer cells, enabling unlimited proliferative capacity [14]. The telomerase holoenzyme is a ribonucleoprotein complex composed of a catalytic subunit with reverse transcriptase activity (TERT), an essential RNA template (TERC), and several auxiliary proteins, including DKC1, NOP10, NHP2, and GAR1, that facilitate assembly, stability, and enzymatic function [15, 16].

In the context of ccRCC, the role of telomere biology is particularly intricate and somewhat paradoxical. Early precancerous lesions and localized tumors often exhibit significant telomere shortening, which suggests a history of replicative stress [17]. However, progression to advanced disease is frequently accompanied by telomerase reactivation, facilitating immortalization and unrestrained growth. Large-scale genome-wide association studies (GWAS) have identified several TL-associated genetic variants that confer susceptibility to various cancers [18, 19]. Nevertheless, establishing a definitive causal link between genetically determined TL and ccRCC risk has proven challenging. This is largely due to methodological limitations inherent in observational epidemiology, such as confounding variables, reverse causation, and tissue-specific telomere dynamics.

To address these challenges, our study integrates population-level genetic methodologies with single-cell transcriptomic analysis to investigate the causal influence of TL on ccRCC development and to delineate its functional repercussions within the tumor microenvironment. First, we employed Mendelian randomization (MR) analyses across several independent cohorts to infer causality between genetically predicted TL and ccRCC risk. Subsequently, these findings were verified through multivariable MR (MVMR) and Bayesian colocalization analyses to account for pleiotropy and ensure that genetic associations were attributable to shared causal variants rather than linkage disequilibrium. Furthermore, we leveraged single-cell RNA sequencing (scRNA-seq) data derived from human ccRCC tumor specimens to dissect the expression patterns of telomerase components across different cell subtypes, assess activation states of cellular senescence pathways, and characterize intercellular communication networks within the tumor ecosystem. We further focused on malignant cells that originate from the proximal tubule epithelium. This integrative strategy not only provides robust genetic evidence supporting a causal role for TL in ccRCC susceptibility but also elucidates novel mechanisms through telomerase-associated genes (such as *NOP10* and *NHP2*). We believe our findings can offer some new insights into the pathogenesis of ccRCC and unveils potential therapeutic targets focused on telomere-related molecular mechanisms.

## 2. Methods and Materials

### 2.1. MR Section

#### 2.1.1. Study Design

**Figure 1:**
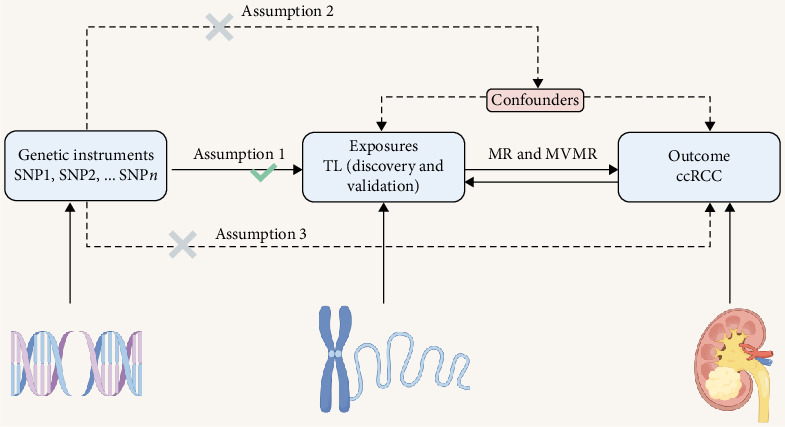
Conceptual framework and assumptions of Mendelian randomization analysis examining causal relationships between TL and ccRcc. ccRCC, clear cell renal carcinoma; IV, instrumental variable; MR, Mendelian randomization; MVMR, multivariable Mendelian randomization; TL, telomere length.

We conducted a bidirectional two-sample MR analysis following the STROBE-MR guidelines (Supporting Information 1: File S1) to investigate the causal relationship between TL and the risk of ccRCC. Genetic instrumental variables for TL were selected from existing large-scale GWAS summary-level data, which included both discovery and validation datasets. We utilized genome-wide significant variants (*p* < 5 × 10^−8^) and implemented stringent linkage disequilibrium filtering (*r*^2^< 0.001, distance > 10,000 kb) to satisfy the three core assumptions of minimum regression analysis: relevance, independence, and exclusion-restriction. The ccRCC outcome data were sourced from the GWAS Catalog. Our analytical framework employed inverse variance weighted (IVW) as the primary method, complemented by various sensitivity analyses (MR–Egger, weighted median, weighted mode, and MR-PRESSO). Additionally, reverse MR was conducted using the same rigorous methodology to assess causality and adjusted for the potential effects of chronic kidney disease (CKD), hypertension, and smoking through multivariable linear regression. We also distinguished between linkage effects and pleiotropy through colocalization analysis. All analyses were performed using R version 4.2.1 with the TwoSampleMR and MR-PRESSO packages, as depicted in Figure 1.

#### 2.1.2. Data Sources

The summary-level GWAS data for TL were derived from the UK Biobank (UKB) cohort, which includes genome-wide genotyping data from 472,174 participants of European ancestry, serving as the discovery set [20]. To enhance the stability of causal inference, we selected validation dataset data from the GWAS Catalog (ID: GCST90435144), which comprises 438,351 individuals of non-Finnish European ancestry [21]. This dataset was chosen based on principal component 1, as it integrates the strengths of two measurement methods, reducing technical noise and better reflecting the true biological variation in TL. Outcomes data for ccRCC were obtained from the GWAS Catalog (ID: GCST90320058), encompassing 752,817 individuals of European ancestry [22]. Potential confounders (including CKD, hypertension, and smoking) were sourced from collaborations, such as FinnGen_R12 [23], UK Biobank (https://opengwas.io/datasets/ukb-a-531), and GSCAN [24], which included 12,787 cases and 480,448 controls for CKD, 337,199 individuals for hypertension, and 249,752 individuals for smoking. To minimize population stratification bias and maintain methodological consistency, we strictly limited the exposure and outcome datasets to individuals of European ancestry, thereby enhancing the reliability of the causal inference analysis. Further details regarding these data sources can be found in Supporting Information 6: Table S1.

#### 2.1.3. Selection of Instrumental Variables

In this study, single nucleotide polymorphisms (SNPs) were used as genetic instrumental variables through a rigorous multistage selection process. Initially, we extracted genome-wide significant variants (*p* < 5 × 10^−8^) from the exposure group's GWAS data, applying this threshold consistently in both forward and reverse analyses. Following this, we implemented linkage disequilibrium filtering (*r*^2^ < 0.001, clustering distance = 10,000 kb) to eliminate potentially confounding correlated variants [25]. We ensured consistency in effect estimates and allele frequencies by coordinating the exposure group with the outcome dataset. The strength of the instrumental variables was evaluated using the *F*-statistic, calculated according to the formula *F* = *R*^2^(*n* – k− 1)*k*(1 − *R*^2^)*F* = *k*(1 − *R*^2^)*R*^2^(*n* – *k*− 1), where variants with *F* < 10*F* < 10 were excluded to maintain sufficient statistical power [26]. We systematically screened the GWAS catalog (https://www.ebi.ac.uk/gwas/) to identify and exclude SNPs significantly associated (*p* < 1 × 10^−5^) with known confounders (including smoking, obesity, hypertension, CKD/dialysis, genetic factors, and occupational exposures), thereby effectively reducing confounding risk. Importantly, validation confirmed that all selected instrumental variables exhibited no significant associations through alternate pathways with potential confounders or outcome variables (Supporting Information 6: Table S3). Detailed characteristics of these genetic instrumental variables are provided in Supporting Information 6: Table S2.

#### 2.1.4. Statistical Analysis

The IVW method was applied as the primary analytical approach. This method offers optimal statistical power by calculating a weighted average of SNP-specific causal estimates, assuming the absence of horizontal pleiotropy [27]. The Cochran *Q* test was performed to assess heterogeneity among exposure-related SNPs: a fixed-effects model was employed in the absence of heterogeneity, while a random-effects model was utilized when heterogeneity was detected [28]. Various complementary methods reinforced the robustness of the findings: MR–Egger regression introduced an intercept term to detect directional pleiotropy, remaining valid under the condition of invalid instrumental variables (*p* < 0.05 indicated the presence of pleiotropy) [29]; the weighted median method maintained consistency even when upto 50% of the statistical weight originated from valid instrumental variables [30]; the weighted mode estimation method identified the distribution characteristics of the weighted SNP-specific causal estimates, making it particularly suitable for analysis under conditions of horizontal pleiotropy [31].

We assessed heterogeneity, pleiotropy, and sensitivity through MR–Egger intercept analysis. An intercept close to zero indicated a low probability of pleiotropy, supplemented by a global test from MR-PRESSO for systematic outlier detection and management [32]. Upon identifying outliers, we employed an iterative optimization process, reperforming MR analyses after their removal to ensure robustness in causal estimates. Sensitivity testing included leave-one-out (LOO) evaluation, systematically excluding single SNPs to examine their influence on overall effect estimates while validating the consistency across different MR methodologies, ensuring that causal inference remained stable and reliable even in the presence of violations of the instrumental variable assumptions [33]. Steiger testing was conducted to verify that the genetic variation serving as instrumental variables was more strongly associated with the exposure variable than with the outcome variable, thereby assuring the integrity of the instrumental variable assumptions and the reliability of the MR study [34].

To assess bidirectional causality, we followed a standardized process for reverse MR analysis, treating ccRCC as the exposure and TL as the outcome variable. All statistical analyses maintained consistent significance thresholds and analytical frameworks across forward and reverse analyses, providing a comprehensive bidirectional validation of the causal relationship between TL and ccRCC.

Univariable genetic estimates evaluated the overall effect of the exposure on the outcome, whereas MVMR decomposed the analysis into independent effects assessments, controlling for confounders, such as CKD, hypertension, and smoking within the same model. The implementation of MVMR employed IVW, multivariable genetic rate-pressure sensitivity analysis (MR-PRESSO), and least absolute shrinkage and selection operator (LASSO) regression techniques. Within the MVMR framework, the IVW method regressed all exposure-associated SNPs against outcomes through inverse variance weighting. To mitigate pleiotropy among instrumental variables, the MR-PRESSO method detected and excluded outliers. Additionally, the LASSO regression technique removed exposure factors exhibiting collinearity.

The coloc R software package was utilized for colocalization analysis. This method assessed the posterior probabilities of five hypotheses (H0, H1, H2, H3, and H4) at each variant locus using a Bayesian framework, identifying common causal variants within specific genomic regions to elucidate the association mechanisms between these factors and the two phenotypes. In particular, the method encompasses five scenarios: (1) no association with either phenotype; (2) associated only with phenotype 1; (3) associated only with phenotype 2; (4) both phenotypes regulated by different causal variants; (5) both phenotypes influenced by common causal variants. Analysis utilized default prior parameters (*p*_1_ = 1 × 10^−4^, *p*_2_ = 1 × 10^−4^, *p*_12_ = 1 × 10^−5^) [35]. When the posterior probability of hypothesis H4 exceeded 80%, it indicated that common causal variants within the genomic region significantly impacted gene expression and ccRCC risk.

### 2.2. scRNA-seq Section

#### 2.2.1. Data Acquisition and Processing

Three scRNA-seq datasets (GSE159115, GSE210038, and GSE237429) [36, 37] were obtained from the Gene Expression Omnibus (GEO) database, including specimens from 12 adjacent normal tissues and 18 tumor tissues of ccRCC. Data preprocessing and downstream analyses were conducted using the “Seurat” package (version 4.4.0) within the R statistical environment. Filtered gene expression matrices were normalized through the “NormalizeData” function. Principal component analysis (PCA) was performed to identify 5000 highly variable genes (HVGs), with 30 principal components selected for subsequent steps. To address batch effects, the “RunHarmony” function from the “harmony” package was implemented. Cell cluster-specific biomarkers were identified utilizing the “wilcoxauc” function from the “presto” package and annotated in reference to the CellMarker 2.0 database [38]. According to canonical biomarkers reported in previous literature, epithelial cells were further classified into the following subpopulations: proximal tubule cells (PTCs), intercalated cells (ICs), ascending limb (AL) cells, principal cells (PCs), descending limb (DL) cells, mesangial cells (MCs), and distal convoluted tubule/connecting tubule (DCT/CNT) cells 1.

#### 2.2.2. Cellular Senescence Evaluation and Trajectory Analysis

Cellular senescence levels were quantified utilizing three validated gene sets associated with senescence, in combination with the “UCell” scoring method [39]. Specifically, the gene sets comprised: 125 human senescence-related genes derived from the SenMayo dataset [40], 525 overexpressed senescence-associated genes curated from CellAge database [41], and a 77-gene signature (FRIDMAN.SENESCENCE.UP) obtained from the Molecular Signatures Database (MSigDB) repository [42]. For inference of cellular progression and differentiation dynamics, pseudotime trajectory reconstruction was performed using the “monocle” package (version 2.26.0), focusing on single-cell transcriptomic profiles of proximal tubule epithelial cells.

#### 2.2.3. Differential Expression Analysis

Tubule epithelial cells were categorized into NOP10-positive and NOP10-negative, as well as NHP2-positive and NHP2-negative populations, based on unique molecular identifier (UMI) counts, where a UMI count greater than zero was classified as positive. The differential expression analysis between biomarker-positive and biomarker-negative tumor cells was conducted using the “Libra” package (version 1.0.0). The “run_de” function was set to the “singlecell” family, employing the Wilcoxon test (with “de_method” specified as “wilcox”). A gene was deemed significantly differentially expressed if its average log fold change (avg_logFC) was greater than 0.25 and the adjusted *p*-value (p_val_adj) was less than 0.05.

#### 2.2.4. Pathway Enrichment Analysis

To explore the critical biological processes associated with NOP10^+^/NHP2^+^ cancer cells, we sourced 50 hallmark gene sets from the MSigDB [43]. For a comprehensive pathway enrichment analysis, we utilized six distinct gene set scoring techniques, including AUCell, UCell, singscore, ssgsea, JASMINE, and viper. The outcomes from these analyses were combined through the robust rank aggregation (RRA) method, applying a significance criterion of RRA score < 0.05. This integration was carried out using the “irGSEA” and “RobustRankAggreg” R packages to effectively reduce any potential biases in the analysis [44, 45].

#### 2.2.5. Intercellular Communication Analysis

Intercellular communication among cell populations within the tumor microenvironment was systematically investigated using the “CellChat” R package (version 2.2.0). This tool infers potential signaling interactions based on curated ligand–receptor pairs, enabling the identification and quantification of cell–cell communication networks. The analysis was conducted using default parameters, and included steps of data preprocessing, construction of signaling networks, and visualization of communication patterns between different cell types.

## 3. Results

### 3.1. MR Section

#### 3.1.1. Two-Sample MR and Reverse MR

**Figure 2:**
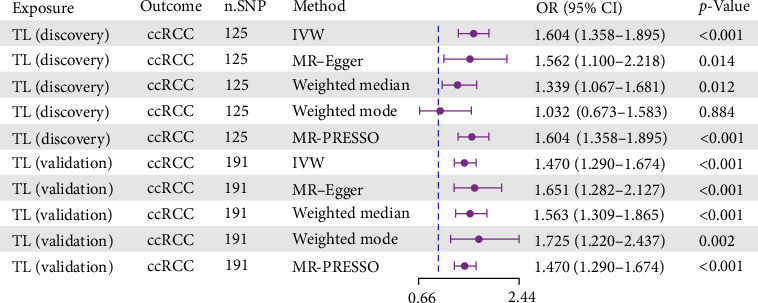
Causal effects of TL on ccRCC risk: two-sample MR analysis. ccRCC, clear cell renal carcinoma; CI, confidence interval; IVW, inverse-variance weighted; MR, Mendelian randomization; n.SNP, number of single nucleotide polymorphisms; OR: odds ratio; TL, telomere length.

Through genome-wide association analysis (*p* < 5 × 10^−8^), we identified significant associations between TL and ccRCC risk in both discovery and validation cohorts. The genetic instruments demonstrated robust statistical power, with *F*-values ranging from 26.18 to 1000.77, substantially exceeding the traditional weak instrument bias threshold (Supporting Information 6: Table S2). TL consistently exhibited significant associations with ccRCC risk across both cohorts (Figure 2). The primary analysis using IVW method revealed a positive correlation between TL and ccRCC risk in the discovery set (odds ratio [OR]: 1.604, 95% confidence interval [CI]: 1.358–1.895, *p* < 0.001). This finding was consistently supported by MR–Egger, weighted median, and MR-PRESSO methods, with the weighted mode approach showing a consistent directional trend, albeit without statistical significance. The validation cohort corroborated these results (OR: 1.470, 95% CI: 1.290–1.674, *p* < 0.001), with alternative methods similarly demonstrating significant positive correlations. The main results are shown in Supporting Information 6: Table S4.

Sensitivity analyses validated the robustness of our study findings. Standard pleiotropy assessments and MR-PRESSO analyses revealed no evidence of horizontal pleiotropy (Supporting Information 6: Table S5), further confirming the reliability of our instrumental variables. Heterogeneity testing demonstrated no significant differences among genetic instruments (Supporting Information 6: Table S6), while LOO analysis confirmed that the observed associations were not influenced by any critical outliers (Supporting Information 2: Figure S1). Steiger directionality tests consistently supported the hypothesized causal relationship, with all instrumental variables passing directional assessment criteria (Supporting Information 6: Table S7). These comprehensive sensitivity analyses collectively reinforced the reliability of the causal inference framework, demonstrating from multiple dimensions that our research results were not substantially interfered with by factors violating MR assumptions such as pleiotropy, heterogeneity, or reverse causality.

**Figure 3:**
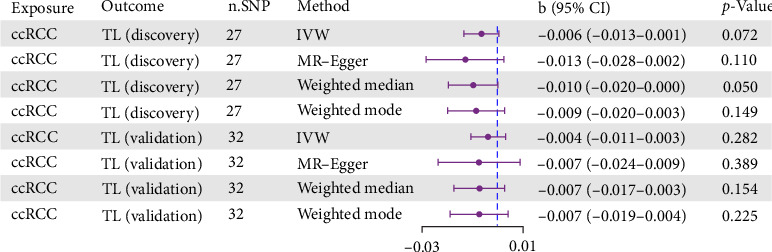
Reverse MR analysis: ccRCC on TL. b, beta value; ccRCC, clear cell renal carcinoma; CI, confidence interval; IVW, inverse-variance weighted; MR, Mendelian randomization; n.SNP, number of single nucleotide polymorphisms; OR, odds ratio; TL, telomere length.

Using consistent SNP selection thresholds with the primary analysis, we conducted a reverse MR analysis and found no evidence of a causal relationship between ccRCC and TL (Figure 3). The primary results and sensitivity analyses, detailed in Supporting Information 6: Tables S4–S7, showed effect estimates approaching zero and consistently failed to achieve statistical significance across different methodological approaches. The absence of a reverse causal relationship strengthens the validity of our forward causal inference, supporting the unidirectional nature of the TL–ccRCC relationship and eliminating concerns about potential bidirectional causal relationships that might have confounded our primary findings. Additional scatter plots and forest plots for these analyses are presented in Supporting Information 3: Figure S2 and Supporting Information 4: Figure S3.

#### 3.1.2. MVMR

**Figure 4:**
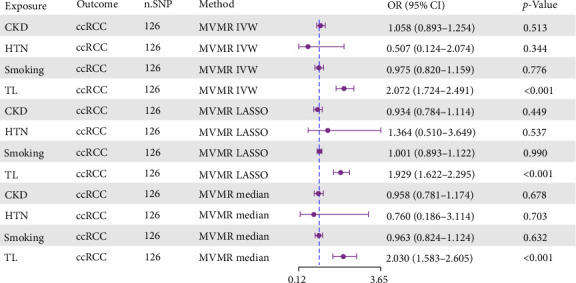
MVMR analysis: independent causal effects of TL on ccRCC risk. ccRCC, clear cell renal carcinoma; CI, confidence interval; CKD, chronic kidney disease; HTN, hypertension; IVW, Inverse-Variance Weighted; MVMR, multivariable Mendelian randomization; n.SNP, number of single nucleotide polymorphisms; OR, odds ratio; TL, telomere length.

To elucidate the potential pleiotropic effects and interactions of TL, we employed three complementary methodological approaches for MVMR analysis: IVW, median, and LASSO regression. Given that CKD, hypertension, and smoking are recognized as major risk factors for ccRCC, we incorporated these three risk factors into the MVMR analysis to mitigate their potential confounding effects on MR results. After adjusting for potential interactions among these risk factors, our analysis demonstrated that TL remained significantly associated with increased ccRCC risk. Using the IVW method, we observed a significant promoting effect (OR: 2.072, 95% CI: 1.724–2.491, *p* < 0.001). Notably, these positive associations persisted when using LASSO regression (OR: 1.929, 95% CI: 1.622–2.295, *p* < 0.001) and median methods (OR: 2.030, 95% CI: 1.583–2.605, *p* < 0.001). The three distinct analytical approaches consistently confirmed the significant promoting effect. Multiple sensitivity analyses validated these findings, providing compelling evidence for the independent causal role of TL in ccRCC development—even when accounting for potential interactions among known risk factors (Supporting Information 6: Table S8 and Figure 4).

#### 3.1.3. Colocalization Analysis

**Figure 5:**
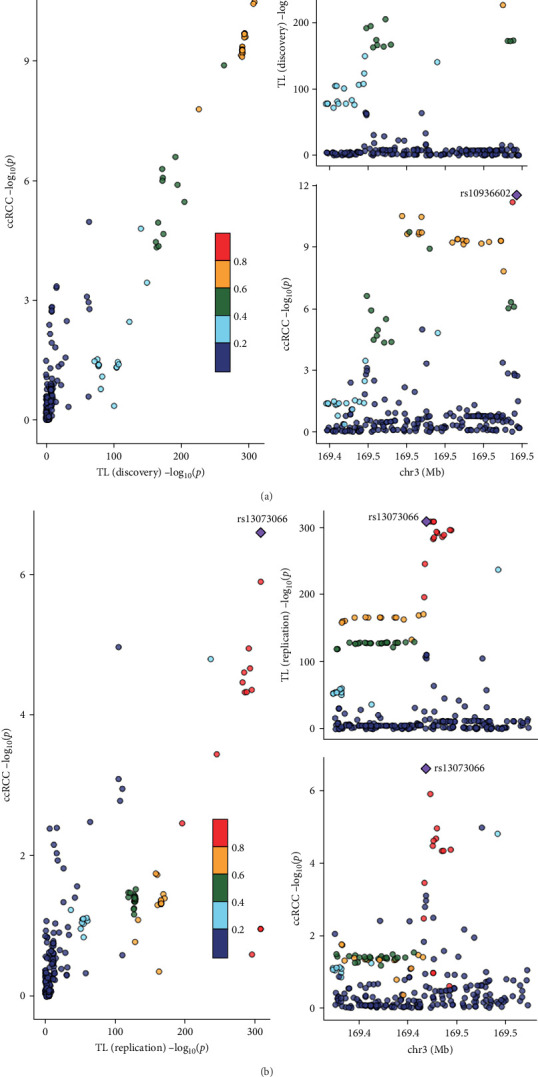
Colocalization analysis results for TL-ccRCC pathways. The pronounced colocalization signals in both (A) and (B) panels demonstrate robust shared genetic architecture between TL and ccRCC risk, with multiple genomic regions displaying high posterior probabilities for common causal variants. (A) TL Discovery to ccRCC: colocalization analysis between telomere length discovery cohort and clear cell renal cell carcinoma risk, showing exceptional evidence for shared causal variants (PP.H4 = 98.3%) with key genomic loci, including rs10936602 displaying strong colocalization signals. (B) TL validation to ccRCC: colocalization analysis between telomere length validation cohort and ccRCC risk, demonstrating near-perfect colocalization evidence (PP.H4 = 99.7%) with rs13073066 and multiple genomic regions confirming shared genetic architecture.

Colocalization analysis provided compelling evidence for shared causal variants between TL and ccRCC (Supporting Information 6: Table S9 and Figure 5). The posterior probabilities for hypothesis H4 (shared causal variants) revealed extraordinarily significant colocalization signals: TL discovery set-ccRCC (PP.H4 = 98.3%) and TL validation set-ccRCC (PP.H4 = 99.7%), both substantially exceeding the robust colocalization threshold of 80%. These findings indicate a shared genetic foundation underlying the causal relationship between TL and ccRCC risk.

### 3.2. scRNA-seq Section

#### 3.2.1. Cellular Composition and Molecular Features of the ccRCC Tumor Microenvironment

**Figure 6:**
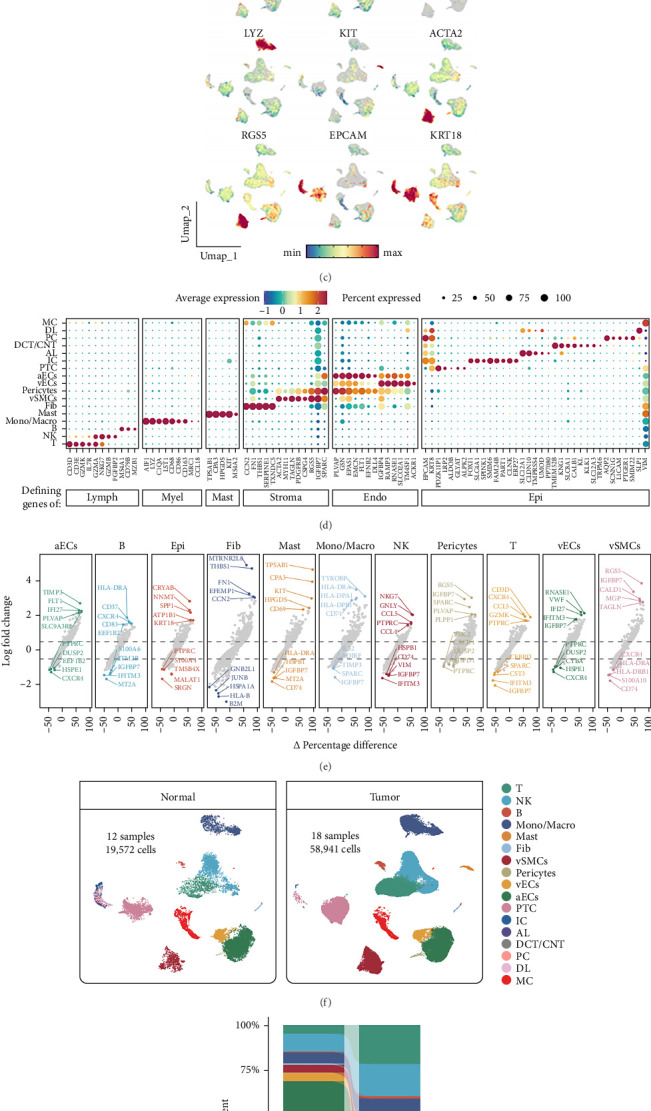
Single-cell transcriptomic profiling in ccRCC microenvironment. (A) Overview of sample composition from three integrated ccRCC single-cell RNA-seq datasets (GSE159115, GSE210038, and GSE237429), comprising 30 samples (18 tumor and 12 adjacent normal tissue). (B) UMAP visualization of 78,513 high-quality single cells from the 30 samples, showing seven epithelial cell types and 10 major stromal and immune cell populations across datasets. Colors indicate cell types, datasets, or individual samples. (C) UMAP plots displaying average expression patterns of canonical marker genes for each major cell cluster. (D) Dot plot representing the percentage of cells expressing key markers per cell type; color intensity corresponds to average expression level. (E) Differential expression analysis of each cluster with the top five up- and downregulated genes highlighted. The *x*-axis indicates the difference in the proportion of expressing cells compared to other clusters. (F) UMAP visualization separated into adjacent normal (left) and tumor (right) samples. (G) Stacked bar plot showing cell type composition proportions in adjacent normal versus tumor samples.

We analyzed a combined cohort of 30 samples, including 18 tumor and 12 adjacent normal tissues, from three publicly available ccRCC single-cell RNA-seq datasets (GSE159115, GSE210038, and GSE237429; Figure 6A). After stringent quality control, 78,513 high-quality cells and 19,122 genes were retained for downstream analysis (Figure 6B). Dimensional reduction and visualization by UMAP revealed clear segregation of major epithelial, immune, and stromal populations. A dot plot of canonical marker genes (Figure 6C,D) allowed robust identification of each cell type: T cells (CD3D, GZMK, and IL7R), NK cells (NKG7, GZMB, and FGFBP2), B cells (MS4A1, CD79B, and MZB1), monocyte/macrophages (LYZ, C1QA, and AIF1), mast cells (TPSAB1, KIT, and CPA3), fibroblasts (CCN2, FN1, and THBS1), vascular and arterial endothelial cells (PLVAP, EMCN, FLT1, EFNB2, and DLL4), and multiple epithelial subpopulations (EPCAM and KRT8). The dot plot illustrates the percentage of cells expressing each marker and the corresponding average expression levels, demonstrating distinct molecular signatures for each cell cluster. Differential expression analysis (Figure 6E) further highlights the unique transcriptomic features of individual cell populations.

Based on previous studies, the epithelial compartment was further delineated into distinct subpopulations, including: AL, characterized by the expression of SLC12A1, CLDN10, TMPRSS4, UMOD, and PP7080; DCT/CNT, marked by TMEM52B, KNG1, SLC8A1, CALB1, KL, KLK1, SLC12A3, and TRPM6; DL, defined by high SLPI expression; ICs, identified by elevated levels of FOXI1, SLC4A1, SPINK1, SMIM6, FAM24B, PART1, CLNK, and ERP27; MCs, delineated by VIM expression; PCs, marked by AQP2, SCNN1G, L1CAM, PTGER1, and SMIM22; PTCs, distinguished by the expression of PDZK1IP1, LRP2, ALDOB, GLYAT, and ALPK2^1^. Fibroblasts are typically distributed within the tumor stroma, but their abundance can vary significantly between samples. In the context of ccRCC, fibroblasts may predominantly express genes such as CCN2, FN1, and THBS1, which are associated with extracellular matrix remodeling and fibrosis. Our single-cell analysis further revealed that ccRCC tumor tissues exhibit a complex cellular composition, characterized by abundant immune cell infiltration and a rich spectrum of vascular cell populations, including arterial and venous endothelial cells (aECs and vECs), vascular smooth muscle cells (vSMCs), and pericytes. This intricate cellular milieu underscores the dynamic interactions within the tumor microenvironment, contributing to the progression and heterogeneity of ccRCC. Comparisons of adjacent normal and tumor samples (Figure 6F) reveal shifts in cellular composition, which are further quantified in a stacked barplot (Figure 6G), collectively illustrating the prominent cellular heterogeneity and dynamic population changes in the ccRCC microenvironment.

#### 3.2.2. Molecular Heterogeneity and Senescence Dynamics of Epithelial Cells in ccRCC

**Figure 7:**
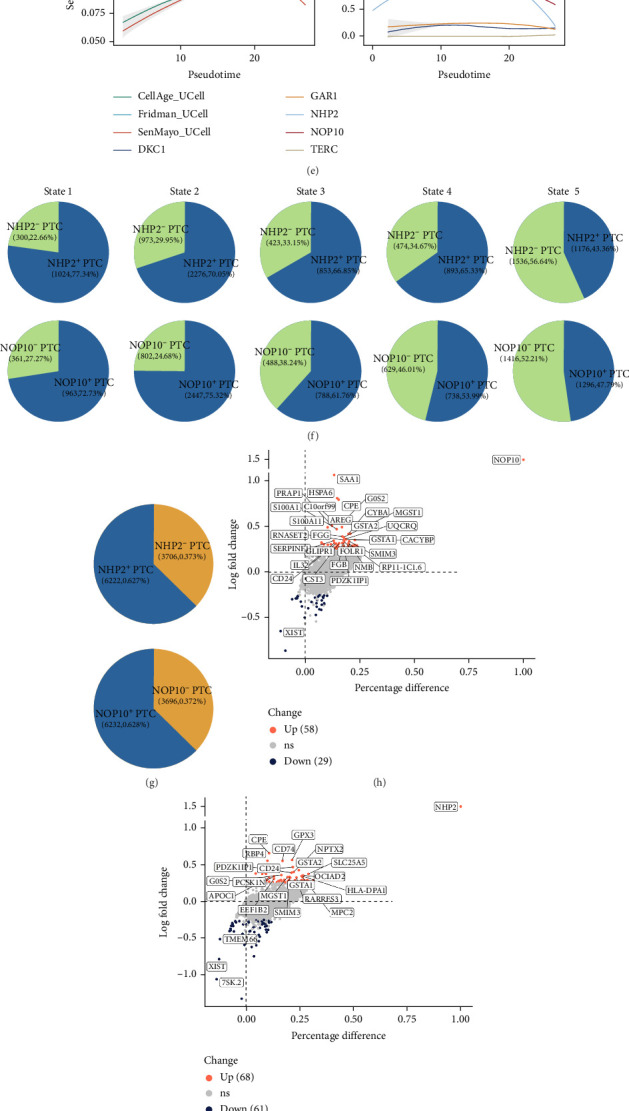
Molecular signatures and functional analyses of epithelial cell subpopulations. (A) Expression patterns of key marker genes across distinct epithelial cell subclusters, including ascending limb (AL), distal convoluted tubule/connecting tubule (DCT/CNT), descending limb (DL), intercalated cell (IC), mesangial cell (MC), principal cell (PC), and proximal tubule Cell (PTC). (B) Violin plots illustrating differences in cellular senescence scores, calculated using UCell with the CellAge, SenMayo, and Fridman senescence-related gene sets, between PTCs derived from normal and tumor tissues. (C) Pseudotime trajectory analysis of PTC cells, depicting state transitions along the differentiation trajectory and (D) state assignments (States 1–5) based on pseudotime progression. (E) Trends of cellular senescence scores along the pseudotime trajectory, including dynamic changes in CellAge_UCell, Fridman_UCell, and SenMayo_UCell signatures, demonstrating fluctuations in senescence levels during state transitions (left panel). The expression trajectories of core telomerase components and associated cofactors across different pseudotime-defined states are shown in the right panel. (F) Pie charts illustrating the proportional distribution of NHP2-positive versus NHP2-negative (upper) and NOP10-positive versus NOP10-negative (lower) tumor cells within each pseudotime-defined state (States 1–5). (G) Overall distribution of NHP2-positive and NHP2-negative (top) and NOP10-positive and NOP10-negative (bottom) proximal tubule cells. (H) Volcano plots highlighting differentially expressed genes between NOP10-positive and NOP10-negative and (I) NHP2-positive and NHP2-negative PTC populations. The *x*-axis denotes the percentage difference in expressing cells between the two groups, while the *y*-axis shows log-transformed fold change. Genes that are significantly upregulated or downregulated are labeled (*p*_val_adj < 0.05, avg_logFC > 0.3). (J) Enrichment analysis of 50 hallmark gene sets comparing NHP2-positive and NHP2-negative PTCs. The *x*-axis represents pathway log fold change, with significance levels indicated by *p*-value and RRA score gradients. Colors distinguish upregulated from downregulated pathways. (K) Equivalent pathway enrichment analysis for NOP10-positive and NOP10-negative PTCs, visualizing pathway activation or suppression along with statistical significance. Statistical significance is indicated as follows: *⁣*^*∗*^*p* < 0.05, *⁣*^*∗∗*^*p* < 0.01, *⁣*^*∗∗∗*^*p* < 0.001, *⁣*^*∗∗∗∗*^*p* < 0.0001, ns = not significant.

Single-cell transcriptomic profiling enabled comprehensive characterization of the epithelial compartment in ccRCC, identifying seven distinct subpopulations, including AL, DCT/CNT, DL, IC, MC, PC, and PTC (Figure 7A). Each epithelial cluster was defined by unique and established marker genes, such as SLC12A1 and UMOD for AL, SLC12A3 and CALB1 for DCT/CNT, SLPI for DL, FOXI1 and SLC4A1 for IC, VIM for MC, AQP2 and SCNN1G for PC, and LRP2 and ALDOB for PTC, which provided high-resolution annotation to map renal segment identities.

Cellular senescence was quantified across epithelial subpopulations using three curated gene sets (CellAge, SenMayo, and Fridman), revealing a marked enrichment of senescence signatures in PTCs (Supporting Information 5: Figures S4), particularly within tumor-derived cells compared to adjacent normal tissues (Figure 7B). To further explore cell state transitions, pseudotime trajectory analysis mapped the developmental progression of PTCs into five discrete stages (States 1‒5; Figure 7C,D). Strikingly, integrated senescence scores demonstrated a nonmonotonic trend along pseudotime, with an initial increase followed by a subsequent decline (Figure 7E, left). This pattern suggests that senescence programs may be activated early in tumor evolution, facilitating antitumor responses and tumor suppression, but are later suppressed as malignant cells acquire the capacity to evade senescence. The expression profiles of telomerase holoenzyme components and associated cofactors were further analyzed. Notably, the majority (TERT, TERC, DKC1, and GAR1) exhibited minimal expression at the single-cell level; however, NOP10 and NHP2 transcripts were readily detectable and highly correlated with senescence states. Specifically, NHP2 expression mirrored senescence dynamics, increasing during early pseudotime stages and declining thereafter, whereas NOP10 demonstrated sustained downregulation across pseudotime, implicating NOP10 as a potential regulator of senescence escape in malignant PTCs (Figure 7E, right). Pie chart analysis (Figure 7F) illustrated a progressive reduction in the proportion of NHP2- and NOP10-positive tumor cells across cell states, supporting these mechanistic insights, and overall ratios were similar between the two markers (Figure 7G). Differential expression analysis between NOP10- or NHP2-positive and -negative PTCs identified key genes (Figure 7H,I). For instance, AREG and FGG were significantly upregulated in NOP10-positive PTCs, genes implicated in epithelial regeneration and immune modulation, while GPX3 and G0S2 were elevated in NHP2-positive PTCs, reflecting enhanced oxidative stress response and metabolic adaptation. Pathway enrichment analysis using hallmark gene sets (Figure 7J,K) revealed robust activation of senescence- and ccRCC-related pathways in NOP10- and NHP2-positive PTCs, including “DNA repair,” “mTORC1 signaling,” “oxidative phosphorylation,” “hypoxia,” “Wnt/β-Catenin signaling,” and “reactive oxygen species pathway”—all critically involved in tumor metabolism, progression, and the establishment or escape of cellular senescence. Collectively, these results illustrate the intricate molecular diversity and dynamic senescence responses within the renal epithelial landscape, highlight the pivotal role of PTCs in ccRCC biology, and uncover NOP10 and NHP2 as novel candidates for functional investigation of senescence and tumor evolution.

#### 3.2.3. Intercellular Communication Patterns of NOP10/NHP2-Positive PTCs in the ccRCC Microenvironment

**Figure 8:**
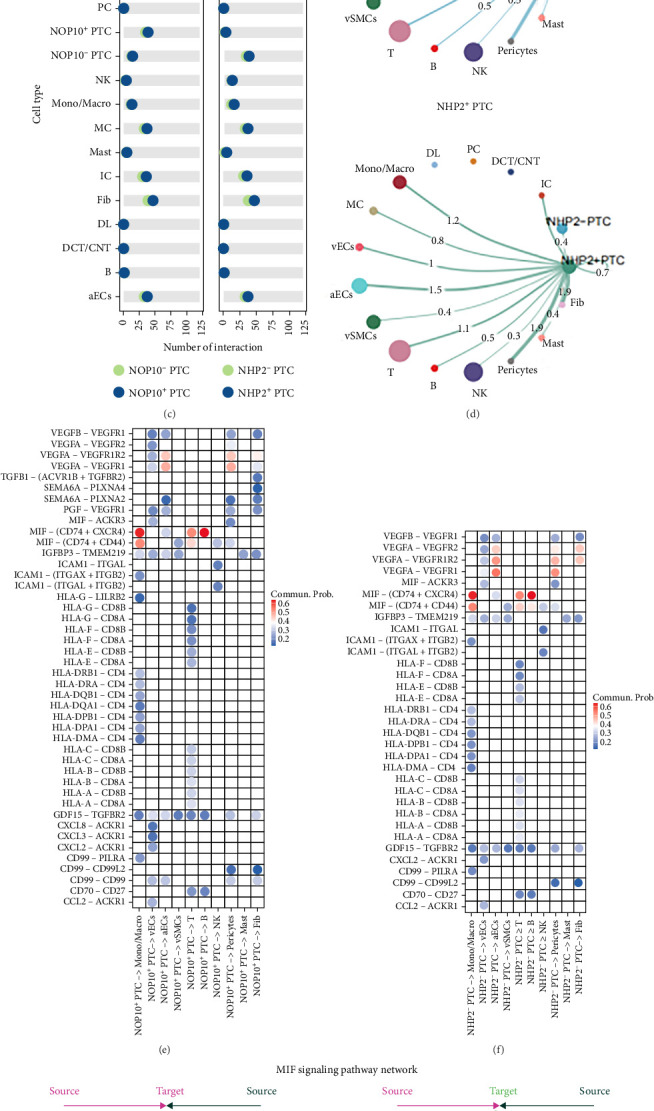
Intercellular communication landscape of NOP10/NHP2-positive PTCs. (A) Distribution of NOP10-positive and NOP10-negative proximal tubule cells (PTCs) across ccRCC samples. (B) UMAP plots showing the expression levels of key signaling pathways, including DNA repair, reactive oxygen species (ROS), and mTORC1 signaling, illustrating cellular distribution and density. (C) Intercellular communication analysis showing the number of ligand–receptor interactions between NOP10-positive and NOP10-negative PTCs (left panel), and between NHP2-positive and NHP2-negative PTCs (right panel). (D) Communication networks between NOP10-positive PTCs and other cell types in the ccRCC tumor microenvironment (upper panel), and between NHP2-positive and NHP2-negative PTCs (lower panel). The thickness of the lines and numbers represent interaction strength. Heatmaps illustrating selected ligand–receptor interactions between NOP10-positive PTCs and other cell types (E), and between NHP2-positive PTCs and other cell types (F), highlighting key signaling molecules. Visualization of the MIF signaling pathway networks, showing the direction and strength of communication from NOP10-positive (G) or NHP2-positive (H) PTCs to interacting cell types, respectively.

To dissect the functional landscape of PTCs in ccRCC, we compared NOP10- and NHP2-positive and -negative PTC populations based on their single-cell transcriptomic profiles (Figure 8A). NOP10-positive PTCs were widely distributed across tumor samples, revealing a heterogeneous spatial pattern within the epithelial compartment of the tumor microenvironment. UMAP plots of DNA repair, reactive oxygen species (ROS), and mTORC1 signaling scores demonstrated a high degree of spatial overlap among these pathways (Figure 8B), suggesting that PTCs exhibiting elevated expression of NOP10 and NHP2 also display active engagement in DNA repair, oxidative stress responses, and mTORC1 signaling. This spatial concordance implicates these pathways as tightly interconnected with cellular senescence phenotypes and the functional states of PTCs in ccRCC. Intercellular communication analysis using ligand–receptor pairing (Figure 8C) showed that both NOP10-positive and NHP2-positive PTCs exhibit highly similar interaction patterns. Notably, there was a pronounced increase in the number of connections with monocyte/macrophages, arterial endothelial cells, pericytes, and fibroblasts, indicating enhanced cross-talk between these epithelial subtypes and the stromal and immune compartments. The global communication networks (Figure 8D) further highlight strong interaction strengths between NOP10^+^/NHP2^+^ PTCs and these microenvironmental cell types, reinforcing the notion that these populations are actively engaged in shaping the tumor milieu. Detailed analysis of selected ligand–receptor interactions (Figure 8E,F) revealed that both NOP10-positive and NHP2-positive PTCs communicate with lymphocytes and monocyte/macrophages predominantly through the MIF (CD74 + CXCR4) and MIF (CD74 + CD44) pathways, as evidenced by higher communication probabilities. In addition, significant interaction strength via VEGFA-mediated signaling was observed between these PTC subsets and arterial endothelial, pericyte, and fibroblast populations, pointing to their potential roles in modulating angiogenesis and stromal remodeling within the tumor ecosystem. In addition, focused mapping of the MIF signaling pathway (Figure 8G,H) demonstrated that both NOP10-positive and NHP2-positive PTCs predominantly send MIF signals to monocyte/macrophages and T cell populations, with a subset of signals received by stromal compartments such as endothelial cells and pericytes. These findings underscore the central role of MIF-mediated intercellular communication by senescent-like epithelial cells in orchestrating immune and stromal crosstalk in the ccRCC microenvironment.

## 4. Discussion

This study integrates genetic epidemiology with single-cell analysis to systematically investigate the causal role of TL in ccRCC and to delineate its functional implications within the TME of ccRCC. Our findings provide robust evidence that genetically determined longer TL is causally associated with an increased risk of ccRCC, and we further identify the telomerase-associated genes, such as *NOP10* and *NHP2* as key regulators of cellular senescence and intercellular communication in ccRCC.

The MR analyses across multiple cohorts consistently demonstrated a positive causal effect of TL on ccRCC risk. The use of independent discovery and validation sets, along with rigorous sensitivity analyses, strengthens the validity of our findings. The absence of reverse causality in the reverse MR analysis further supports the directionality of this relationship. These results are consistent with previous GWAS that have linked TL-associated genetic variants to various cancers, yet they provide stronger causal inference by mitigating confounding and reverse causation biases inherent in observational studies [46]. The MVMR analysis, which adjusted for CKD, hypertension, and smoking—established risk factors for ccRCC—confirmed that the effect of TL on ccRCC risk is independent of these confounders.

The colocalization analysis provided further genetic evidence by demonstrating that TL and ccRCC share common causal variants in specific genomic regions. The high posterior probabilities (PP.H4 > 98%) indicate that the genetic association between TL and ccRCC is likely driven by shared causal mechanisms rather than linkage disequilibrium. This reinforces the notion that telomere maintenance is intrinsically linked to ccRCC development at the genetic level.

To bridge population-level findings with cellular mechanisms, we turned to single-cell transcriptomics. Our analysis of scRNA-seq data from human ccRCC samples revealed that PTCs, the cell type of origin for ccRCC, exhibit pronounced heterogeneity in senescence states. Using multiple senescence gene sets, we found that PTCs from tumor tissues display elevated senescence scores compared to normal tissues. Our trajectory analysis revealed a rise and fall in cellular senescence over time. We propose that this senescence acts as an initial barrier to tumor development, but that cancer cells eventually evade this mechanism to enable progression.

Notably, among the core telomerase components, NOP10 and NHP2 were detectable at the single-cell level and showed strong correlations with senescence dynamics. *NHP2* expression mirrored the rise and fall of senescence scores along pseudotime, while *NOP10* was consistently downregulated in tumor cells, implying its potential role in senescence escape. Differential expression and pathway enrichment analyses revealed that NOP10- and NHP2-positive PTCs are enriched in pathways critical to cancer biology, including DNA repair, mTORC1 signaling, oxidative phosphorylation, hypoxia, and ROS response. These pathways are known to be involved in metabolic reprograming, stress adaptation, and evasion of growth suppression—hallmarks of cancer [47, 48].

Moreover, intercellular communication analysis revealed that NOP10^+^ and NHP2^+^ PTCs exhibit enhanced interactions with stromal and immune cells, particularly via MIF- and VEGFA-mediated signaling. These findings suggest that senescent-like PTCs may actively remodel the tumor microenvironment by promoting angiogenesis, fibrosis, and immune modulation. The MIF pathway, in particular, has been implicated in tumor inflammation and immune evasion, and its activation in NOP10^+^/NHP2^+^ PTCs may facilitate cross-talk with macrophages and T cells, contributing to an immunosuppressive niche in the ccRCC TME [49, 50].

We acknowledge that our study has several limitations. First, while MR supports causality, the genetic instruments for TL are derived from leukocyte TL, which may not fully reflect telomere dynamics in renal tissue. Second, functional experiments are needed to validate the roles of NOP10 and NHP2 in senescence and ccRcc progression. Third, the sample size, though substantial, may still limit the detection of rare cell states or subtle expression changes.

Despite these limitations, our integrative approach provides a comprehensive framework linking genetic predisposition to cellular behavior in ccRCC. We propose a model wherein longer TL increases ccRCC risk by promoting telomere stability in pre-malignant cells, facilitating clonal expansion. During tumor evolution, telomerase reactivation—possibly modulated by NOP10 and NHP2—enables cells to evade senescence and acquire malignant traits. These cells then engage in active cross-talk with the microenvironment to support tumor growth and immune evasion.

In conclusion, our study offers multilevel evidence that supports the causal role of TL in ccRCC and highlights the functional importance of some key telomerase-associated genes in regulating cellular senescence and cellular communication. These insights not only advance our understanding of ccRCC pathogenesis but also identify potential therapeutic targets aimed at telomere maintenance and senescence pathways.

## 5. Conclusion

Our integrative study bridges genetic epidemiology with cellular mechanisms to comprehensively investigate the causal relationship between TL and ccRcc. By combining MR across multiple cohorts with single-cell transcriptomics, we provide robust evidence that genetically determined longer TL causally increases ccRCC risk. Our findings not only confirm population-level genetic associations but also elucidate the intricate cellular processes underlying this relationship. Specifically, we reveal how PTCs with telomerase-associated genes, like *NOP10* and *NHP2* dynamically modulate cellular senescence and interact with the tumor microenvironment. These insights suggest that telomere maintenance plays a critical role in ccRCC development, potentially through mechanisms involving senescence evasion, metabolic reprograming, and complex intercellular communication. While further functional validation is needed, our study offers a comprehensive framework for understanding ccRCC pathogenesis and identifies promising molecular targets for future therapeutic interventions.

## Data Availability

The datasets used in this study are publicly available. The telomere length (TL) GWAS summary statistics were derived from the UK Biobank cohort and the GWAS Catalog. Clear cell renal cell carcinoma (ccRCC) outcome data were obtained from the GWAS Catalog (ID: GCST90320058). Potential confounding factors were sourced from FinnGen_R12, UK Biobank, and GSCAN. The single-cell RNA sequencing (scRNA-seq) datasets were retrieved from the Gene Expression Omnibus (GEO) database, specifically the datasets GSE159115, GSE210038, and GSE237429. These datasets are freely accessible in their respective public repositories. All data and code used in this study can be obtained from the corresponding author upon reasonable request.

## Supporting Information

Supporting Information 1 File S1. STROBE checklist.

Supporting Information 2 Figure S1. Leave-one-out (LOO) sensitivity analysis results.

Supporting Information 3 Figure S2. Scatter plots.

Supporting Information 4 Figure S3. Forest plots.

Supporting Information 5 Figure S4. DNA-binding motif analysis and base stacking patterns for NOP10 and NHP2 regulatory regions.

Supporting Information 6 Table S1. Data source details containing information about the GWAS datasets used in the study. Table S2. Detailed characteristics of genetic instrumental variables, including *F*-statistics. Table S3. Validation confirming that selected instrumental variables show no significant associations with potential confounders or outcome variables. Table S4. Main results of two-sample MR analysis showing causal effects of telomere length on ccRCC risk. Table S5. Standard pleiotropy assessments and MR-PRESSO analysis results showing no evidence of horizontal pleiotropy. Table S6. Heterogeneity testing results demonstrating no significant differences among genetic instruments. Table S7. Steiger directionality test results supporting the hypothesized causal relationship. Table S8. Multiple sensitivity analyses for multivariable MR (MVMR) findings. Table S9: Colocalization analysis results showing shared causal variants between TL and ccRCC.
