# Supplementary material for: Telomere Length and Clear Cell Renal Cell Carcinoma: Unraveling Causal Mechanisms Through Integrative Genetic and Single-Cell Transcriptomic Analysis
Source: Mediators Inflamm. 2025 Nov 27;2025:3705788. doi: 10.1155/mi/3705788 (PMC12677994; doi:10.1155/mi/3705788)
Supplement: Supporting Information 1 — File S1. STROBE checklist. [file 3705788.f1.docx]

**STROBE-MR checklist of recommended items to address in reports of Mendelian randomization studies**^1^ ^2^

| **Item No.** | **Section** | **Checklist item** | **Page No.** | **Relevant text from manuscript** |
| --- | --- | --- | --- | --- |
| 1 | **TITLE and ABSTRACT** | Indicate Mendelian randomization (MR) as the study’s design in the title and/or the abstract if that is a main purpose of the study | 1 | Telomere Length and Clear Cell Renal Cell Carcinoma: Unraveling Causal Mechanisms through Integrative Genetic and Single-Cell Transcriptomic Analysis |
|  | **INTRODUCTION** |  |  |  |
| 2 | **Background** | Explain the scientific background and rationale for the reported study. What is the exposure? Is a potential causal relationship between exposure and outcome plausible? Justify why MR is a helpful method to address the study question | 2-4 | Clear cell renal cell carcinoma (ccRCC) is the most prevalent and aggressive histological subtype of renal cancer.... |
| 3 | **Objectives** | State specific objectives clearly, including pre-specified causal hypotheses (if any). State that MR is a method that, under specific assumptions, intends to estimate causal effects | 3-4 | we employed Mendelian randomization (MR) analyses across several independent cohorts to infer causality between genetically predicted TL and ccRCC risk. Subsequently, these findings were verified through multivariable MR and Bayesian colocalization analyses to account for pleiotropy and ensure that genetic associations were attributable to shared causal variants rather than linkage disequilibrium. |
|  | **METHODS** |  | 4-5 | We conducted a bidirectional two-sample(MR analysis following the STROBE-MR guidelines to investigate the causal relationship between TL and the risk of ccRCC..... |
| 4 | **Study design and data sources** | Present key elements of the study design early in the article. Consider including a table listing sources of data for all phases of the study. For each data source contributing to the analysis, describe the following: |  |  |
|  | a) | Setting: Describe the study design and the underlying population, if possible. Describe the setting, locations, and relevant dates, including periods of recruitment, exposure, follow-up, and data collection, when available. | 4-5 | Genetic instrumental variables for TL were selected from existing large-scale GWAS summary-level data, which included both discovery and validation datasets... |
|  | b) | Participants: Give the eligibility criteria, and the sources and methods of selection of participants. Report the sample size, and whether any power or sample size calculations were carried out prior to the main analysis | 5 | The summary-level GWAS data for TL were derived from the UK Biobank (UKB) cohort, which includes genome-wide genotyping data from 472,174 participants of European ancestry, serving as the discovery set... |
|  | c) | Describe measurement, quality control and selection of genetic variants | 4-5 | We utilized genome-wide significant variants (P<5×10^-8) and implemented stringent linkage disequilibrium filtering (r²<0.001, distance>10,000 kb) to satisfy the three core assumptions of minimum regression analysis: relevance, independence, and exclusion-restriction |
|  | d) | For each exposure, outcome, and other relevant variables, describe methods of assessment and diagnostic criteria for diseases | 5 | Outcomes data for ccRCC were obtained from the GWAS Catalog (ID: GCST90320058), encompassing 752,817 individuals of European ancestry |
|  | e) | Provide details of ethics committee approval and participant informed consent, if relevant |  | NA |
| 5 | **Assumptions** | Explicitly state the three core IV assumptions for the main analysis (relevance, independence and exclusion restriction) as well assumptions for any additional or sensitivity analysis | 4 | We utilized genome-wide significant variants (P<5×10^-8) and implemented stringent linkage disequilibrium filtering (r²<0.001, distance>10,000 kb) to satisfy the three core assumptions of minimum regression analysis: relevance, independence, and exclusion-restriction. . |
| 6 | **Statistical methods: main analysis** | Describe statistical methods and statistics used |  |  |
|  | a) | Describe how quantitative variables were handled in the analyses (i.e., scale, units, model) | 5-6 | single nucleotide polymorphisms (SNPs) were used as genetic instrumental variables through a rigorous multi-stage selection process. Initially, we extracted genome-wide significant variants (P<5×10^-8) from the exposure group’s GWAS datas. ... |
|  | b) | Describe how genetic variants were handled in the analyses and, if applicable, how their weights were selected | 6 | We ensured consistency in effect estimates and allele frequencies by coordinating the exposure group with the outcome dataset. The strength of the instrumental variables was evaluated using the F-statistic, calculated according to the formula F=R2(n−k−1)k(1−R2)F=k(1−R2)R2(n−k−1)​, where variants with F<10F<10 were excluded to maintain sufficient statistical power. |
|  | c) | Describe the MR estimator (e.g. two-stage least squares, Wald ratio) and related statistics. Detail the included covariates and, in case of two-sample MR, whether the same covariate set was used for adjustment in the two samples | 6-7 | The inverse variance weighting (IVW) method was applied as the primary analytical approach. This method offers optimal statistical power by calculating a weighted average of SNP-specific causal estimates, assuming the absence of horizontal pleiotropy... |
|  | d) | Explain how missing data were addressed |  | na |
|  | e) | If applicable, indicate how multiple testing was addressed |  | na |
| 7 | **Assessment of assumptions** | Describe any methods or prior knowledge used to assess the assumptions or justify their validity | 3 | In the context of ccRCC, the role of telomere biology is particularly intricate and somewhat paradoxical. Early precancerous lesions and localized tumors often exhibit significant telomere shortening, which suggests a history of replicative stress..... |
| 8 | **Sensitivity analyses and additional analyses** | Describe any sensitivity analyses or additional analyses performed (e.g. comparison of effect estimates from different approaches, independent replication, bias analytic techniques, validation of instruments, simulations) | 6 | The Cochran Q test was performed to assess heterogeneity among exposure-related SNPs: a fixed-effects model was employed in the absence of heterogeneity, while a random-effects model was utilized when heterogeneity was detected.... |
| 9 | **Software and pre-registration** |  |  |  |
|  | a) | Name statistical software and package(s), including version and settings used | 5 | All analyses were performed using R version 4.2.1 with the TwoSampleMR and MR-PRESSO packages |
|  | b) | State whether the study protocol and details were pre-registered (as well as when and where) |  | NA |
|  | **RESULTS** |  |  |  |
| 10 | **Descriptive data** |  |  |  |
|  | a) | Report the numbers of individuals at each stage of included studies and reasons for exclusion. Consider use of a flow diagram | 5 | as depicted in Figure 1. |
|  | b) | Report summary statistics for phenotypic exposure(s), outcome(s), and other relevant variables (e.g. means, SDs, proportions) | 10 | Through genome-wide association analysis (P<5×10⁻⁸), we identified significant associations between TL and ccRCC risk in both discovery and validation cohorts. The genetic instruments demonstrated robust statistical power, with F-values ranging from 26.18 to 1000.77, substantially exceeding the traditional weak instrument bias threshold |
|  | c) | If the data sources include meta-analyses of previous studies, provide the assessments of heterogeneity across these studies |  | NA |
|  | d) | For two-sample MR:  i.  Provide justification of the similarity of the genetic variant-exposure associations between the exposure and outcome samples  ii.  Provide information on the number of individuals who overlap between the exposure and outcome studies |  | NA |
| 11 | **Main results** |  |  |  |
|  | a) | Report the associations between genetic variant and exposure, and between genetic variant and outcome, preferably on an interpretable scale | 10 | TL consistently exhibited significant associations with ccRCC risk across both cohorts (Figure 2). The primary analysis using IVW method revealed a positive correlation between TL and ccRCC risk in the discovery set (odds ratio [OR]: 1.604, 95% confidence interval [CI]: 1.358-1.895, p < 0.001)., |
|  | b) | Report MR estimates of the relationship between exposure and outcome, and the measures of uncertainty from the MR analysis, on an interpretable scale, such as odds ratio or relative risk per SD difference | 10 | The primary analysis using IVW method revealed a positive correlation between TL and ccRCC risk in the discovery set (odds ratio [OR]: 1.604, 95% confidence interval [CI]: 1.358-1.895, p < 0.001)... |
|  | c) | If relevant, consider translating estimates of relative risk into absolute risk for a meaningful time period |  | NA |
|  | d) | Consider plots to visualize results (e.g. forest plot, scatterplot of associations between genetic variants and outcome versus between genetic variants and exposure) | 10-11 | TL consistently exhibited significant associations with ccRCC risk across both cohorts (Figure 2). . |
| 12 | **Assessment of assumptions** |  |  |  |
|  | a) | Report the assessment of the validity of the assumptions | 10-11 | Sensitivity analyses validated the robustness of our study findings. Standard pleiotropy assessments and MR-PRESSO analyses revealed no evidence of horizontal pleiotropy (Supplementary Table S5)... |
|  | b) | Report any additional statistics (e.g., assessments of heterogeneity across genetic variants, such as *I^2^*, Q statistic or E-value) | 11 | Heterogeneity testing demonstrated no significant differences among genetic instruments (Supplementary Table S6). |
| 13 | **Sensitivity analyses and additional analyses** |  |  |  |
|  | a) | Report any sensitivity analyses to assess the robustness of the main results to violations of the assumptions | 10-11 | Sensitivity analyses validated the robustness of our study findings. Standard pleiotropy assessments and MR-PRESSO analyses revealed no evidence of horizontal pleiotropy. |
|  | b) | Report results from other sensitivity analyses or additional analyses | 10-11 | Sensitivity analyses validated the robustness of our study findings. ... |
|  | c) | Report any assessment of direction of causal relationship (e.g., bidirectional MR) | 11 | Steiger directionality tests consistently supported the hypothesized causal relationship, with all instrumental variables passing directional assessment criteria (Supplementary Table S7). |
|  | d) | When relevant, report and compare with estimates from non-MR analyses |  | NA |
|  | e) | Consider additional plots to visualize results (e.g., leave-one-out analyses) | 11 | while LOO analysis confirmed that the observed associations were not influenced by any critical outliers (Supplementary Figure S1). |
|  | **DISCUSSION** |  | 16 | This study integrates genetic epidemiology with single-cell analysis to systematically investigate the causal role of TL in ccRCC and to delineate its functional implications within the TME of ccRCC. ... |
| 14 | **Key results** | Summarize key results with reference to study objectives | 17 | The MR analyses across multiple cohorts consistently demonstrated a positive causal effect of TL on ccRCC risk. The use of independent discovery and replication sets, along with rigorous sensitivity analyses, strengthens the validity of our findings.... |
| 15 | **Limitations** | Discuss limitations of the study, taking into account the validity of the IV assumptions, other sources of potential bias, and imprecision. Discuss both direction and magnitude of any potential bias and any efforts to address them | 18 | We acknowledge that our study has several limitations. First, while MR supports causality, the genetic instruments for TL are derived from leukocyte telomere length, which may not fully reflect telomere dynamics in renal tissue. .... |
| 16 | **Interpretation** |  |  |  |
|  | a) | Meaning: Give a cautious overall interpretation of results in the context of their limitations and in comparison with other studies | 17 | The MVMR analysis, which adjusted for chronic kidney disease, hypertension, and smoking—established risk factors for ccRCC—confirmed that the effect of TL on ccRCC risk is independent of these confounders. |
|  | b) | Mechanism: Discuss underlying biological mechanisms that could drive a potential causal relationship between the investigated exposure and the outcome, and whether the gene-environment equivalence assumption is reasonable. Use causal language carefully, clarifying that IV estimates may provide causal effects only under certain assumptions | 18 | intercellular communication analysis revealed that NOP10⁺ and NHP2⁺ PTCs exhibit enhanced interactions with stromal and immune cells, particularly via MIF- and VEGFA-mediated signaling. These findings suggest that senescent-like PTCs may actively remodel the tumor microenvironment by promoting angiogenesis, fibrosis, and immune modulation. The MIF pathway, in particular, has been implicated in tumor inflammation and immune evasion, and its activation in NOP10⁺/NHP2⁺ PTCs may facilitate cross-talk with macrophages and T cells, contributing to an immunosuppressive niche in the ccRCC TME.... |
|  | c) | Clinical relevance: Discuss whether the results have clinical or public policy relevance, and to what extent they inform effect sizes of possible interventions | 18 | These cells then engage in active cross-talk with the microenvironment to support tumor growth and immune evasion . |
| 17 | **Generalizability** | Discuss the generalizability of the study results (a) to other populations, (b) across other exposure periods/timings, and (c) across other levels of exposure | 18-19 | These insights not only advance our understanding of ccRCC pathogenesis but also identify potential therapeutic targets aimed at telomere maintenance and senescence pathways. |
|  | **OTHER INFORMATION** |  |  |  |
| 18 | **Funding** | Describe sources of funding and the role of funders in the present study and, if applicable, sources of funding for the databases and original study or studies on which the present study is based | 28 | No funding.. |
| 19 | **Data and data sharing** | Provide the data used to perform all analyses or report where and how the data can be accessed, and reference these sources in the article. Provide the statistical code needed to reproduce the results in the article, or report whether the code is publicly accessible and if so, where | 27 | The datasets used in this study are publicly available. The telomere length (TL) GWAS summary statistics were derived from the UK Biobank cohort and the GWAS Catalog. Clear cell renal cell carcinoma (ccRCC) outcome data were obtained from the GWAS Catalog (ID: GCST90320058). Potential confounding factors were sourced from FinnGen_R12, UK Biobank, and GSCAN. The single-cell RNA sequencing (scRNA-seq) datasets were retrieved from the Gene Expression Omnibus (GEO) database, specifically the datasets GSE159115, GSE210038, and GSE237429. These datasets are freely accessible in their respective public repositories. All data and code used in this study can be obtained from the corresponding author upon reasonable request. |
| 20 | **Conflicts of Interest** | All authors should declare all potential conflicts of interest | 28 | No Competing Interests. |

This checklist is copyrighted by the Equator Network under the Creative Commons Attribution 3.0 Unported (CC BY 3.0) license.

1. Skrivankova VW, Richmond RC, Woolf BAR, Yarmolinsky J, Davies NM, Swanson SA, et al. Strengthening the Reporting of Observational Studies in Epidemiology using Mendelian Randomization (STROBE-MR) Statement. JAMA. 2021;under review.

2. Skrivankova VW, Richmond RC, Woolf BAR, Davies NM, Swanson SA, VanderWeele TJ, et al. Strengthening the Reporting of Observational Studies in Epidemiology using Mendelian Randomisation (STROBE-MR): Explanation and Elaboration. BMJ. 2021;375:n2233.
