# Supplementary material for: Telomere Length and Clear Cell Renal Cell Carcinoma: Unraveling Causal Mechanisms Through Integrative Genetic and Single-Cell Transcriptomic Analysis
Source: Mediators Inflamm. 2025 Nov 27;2025:3705788. doi: 10.1155/mi/3705788 (PMC12677994; doi:10.1155/mi/3705788)
Supplement: Supporting Information 2 — Figure S1. Leave-one-out (LOO) sensitivity analysis results. [file 3705788.f2.pdf]

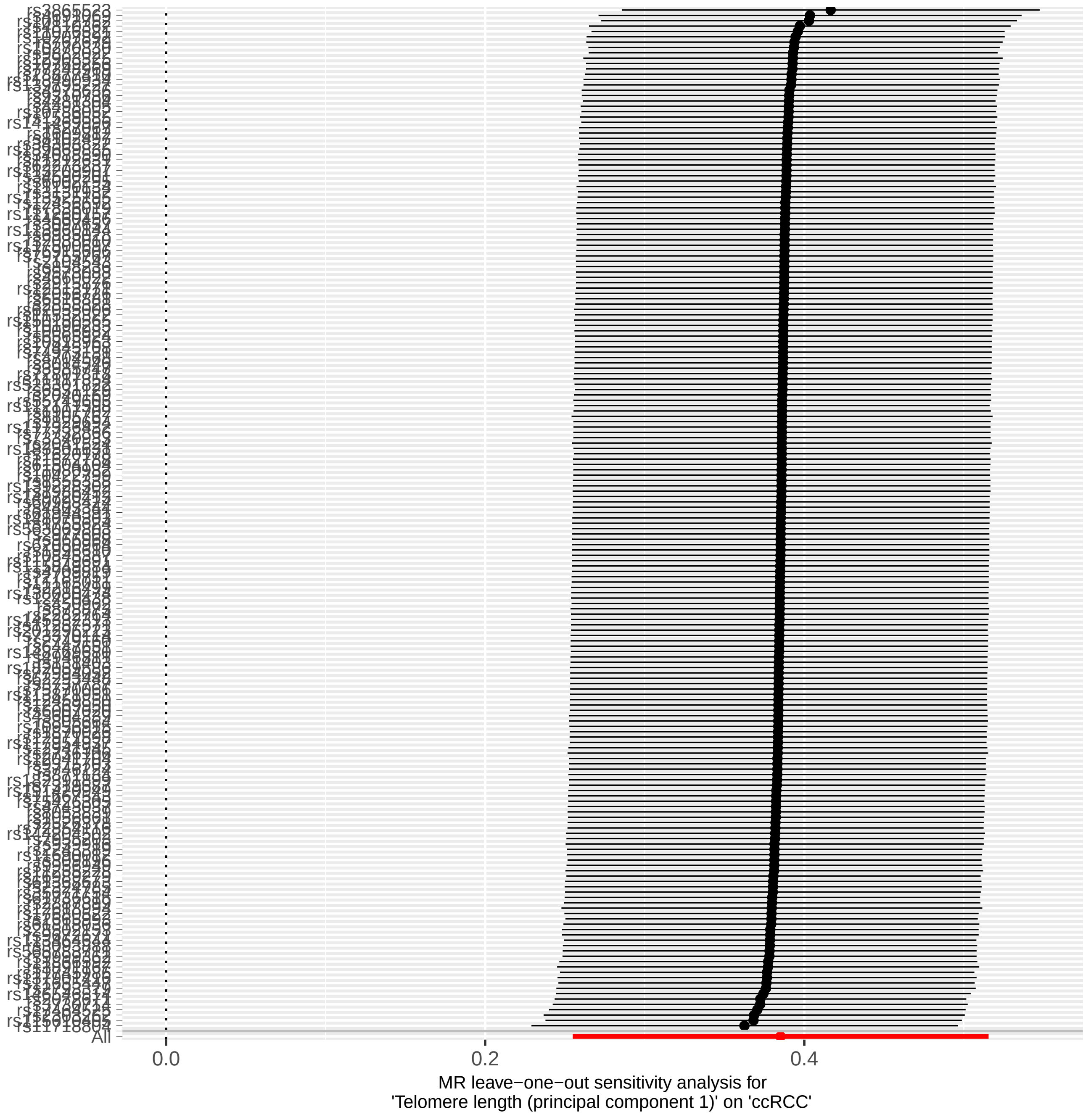
All
0.0
0.2
0.4
MR leave−one−out sensitivity analysis for
'Telomere length (principal component 1)' on 'ccRCC'

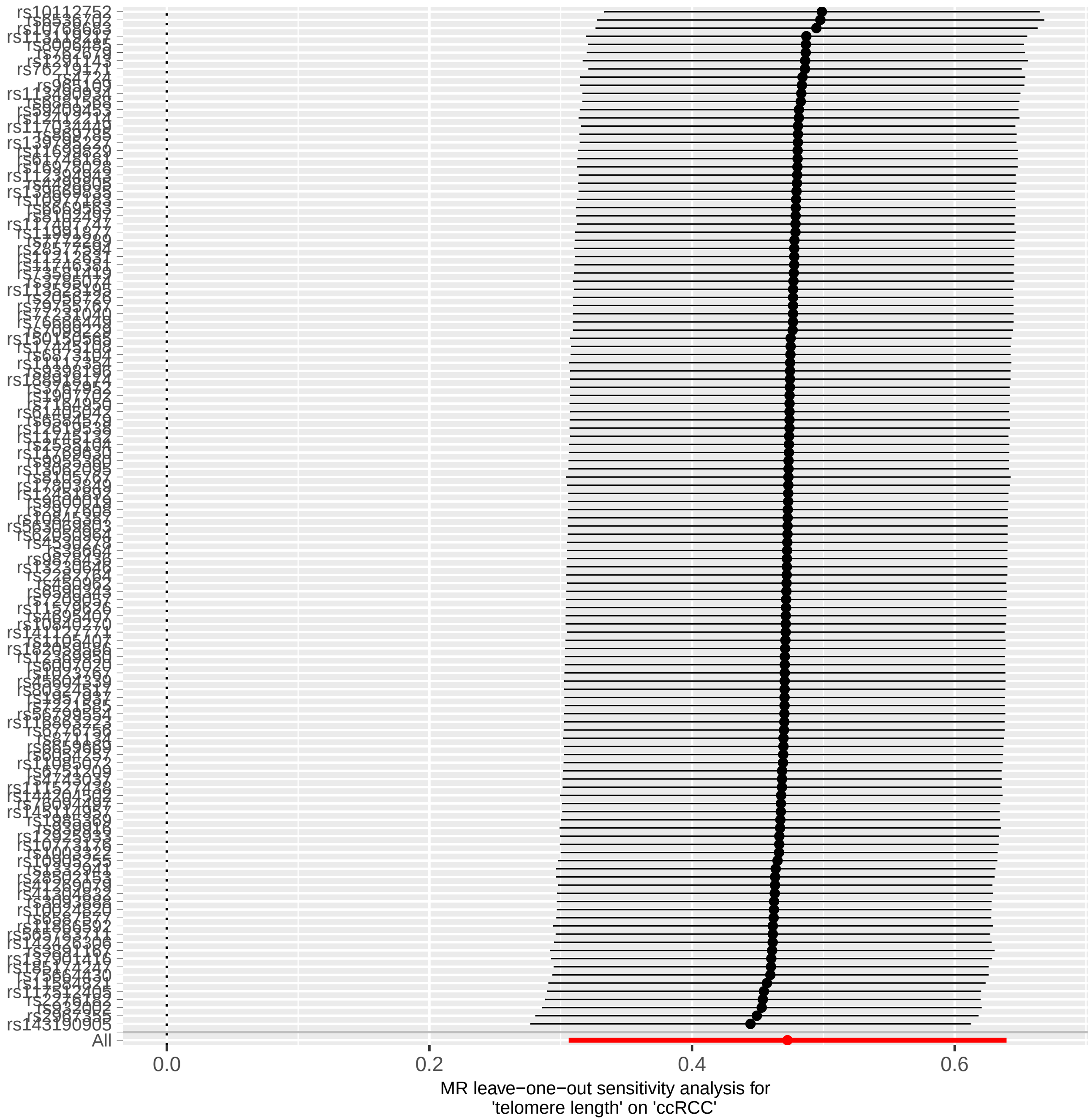

rs10112752
rs143190905
All
0.0
0.2
0.4
0.6
MR leave−one−out sensitivity analysis for
'telomere length' on 'ccRCC'

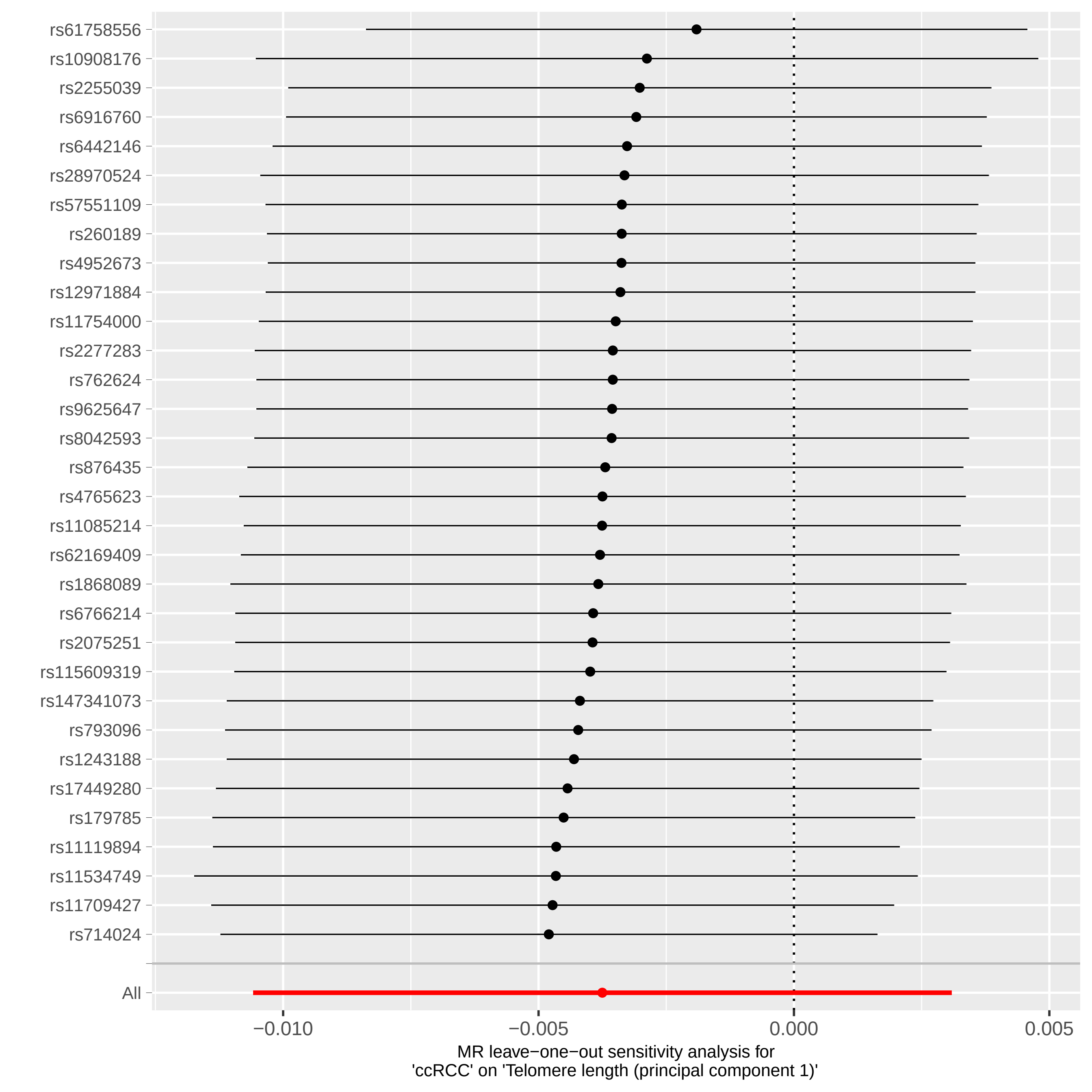
rs61758556
rs10908176
rs2255039
rs6916760
rs6442146
rs28970524
rs57551109
rs260189
rs4952673
rs12971884
rs11754000
rs2277283
rs762624
rs9625647
rs8042593
rs876435
rs4765623
rs11085214
rs62169409
rs1868089
rs6766214
rs2075251
rs115609319
rs147341073
rs793096
rs1243188
rs17449280
rs179785
rs11119894
rs11534749
rs11709427
rs714024
All
−0.010
−0.005
0.000
0.005
MR leave−one−out sensitivity analysis for
'ccRCC' on 'Telomere length (principal component 1)'

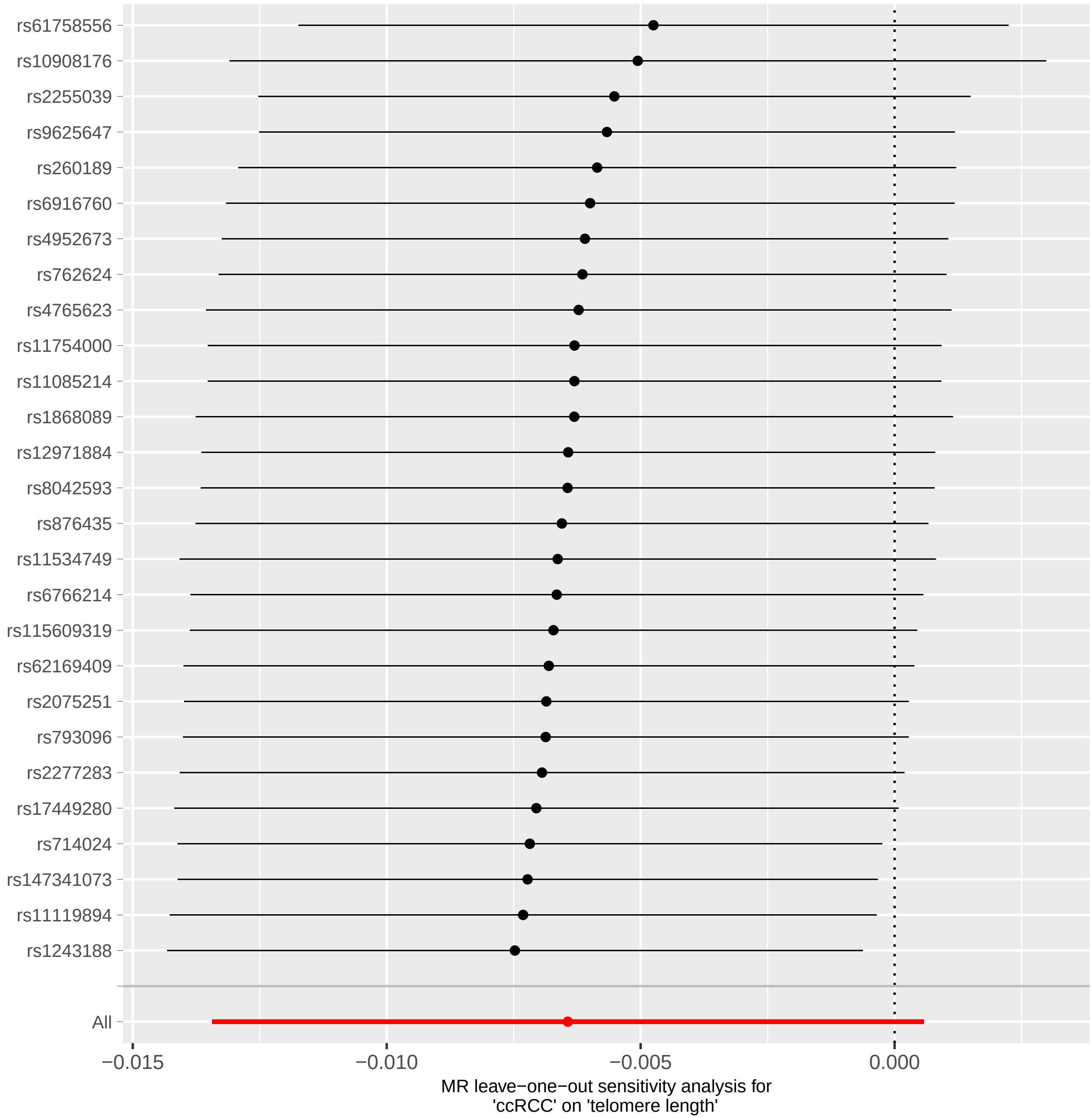
rs61758556
rs10908176
rs2255039
rs9625647
rs260189
rs6916760
rs4952673
rs762624
rs4765623
rs11754000
rs11085214
rs1868089
rs12971884
rs8042593
rs876435
rs11534749
rs6766214
rs115609319
rs62169409
rs2075251
rs793096
rs2277283
rs17449280
rs714024
rs147341073
rs11119894
rs1243188
All
−0.015
−0.010
−0.005
0.000
MR leave−one−out sensitivity analysis for
'ccRCC' on 'telomere length'
