# Supplementary material for: Telomere Length and Clear Cell Renal Cell Carcinoma: Unraveling Causal Mechanisms Through Integrative Genetic and Single-Cell Transcriptomic Analysis
Source: Mediators Inflamm. 2025 Nov 27;2025:3705788. doi: 10.1155/mi/3705788 (PMC12677994; doi:10.1155/mi/3705788)
Supplement: Supporting Information 3 — Figure S2. Scatter plots. [file 3705788.f3.pdf]

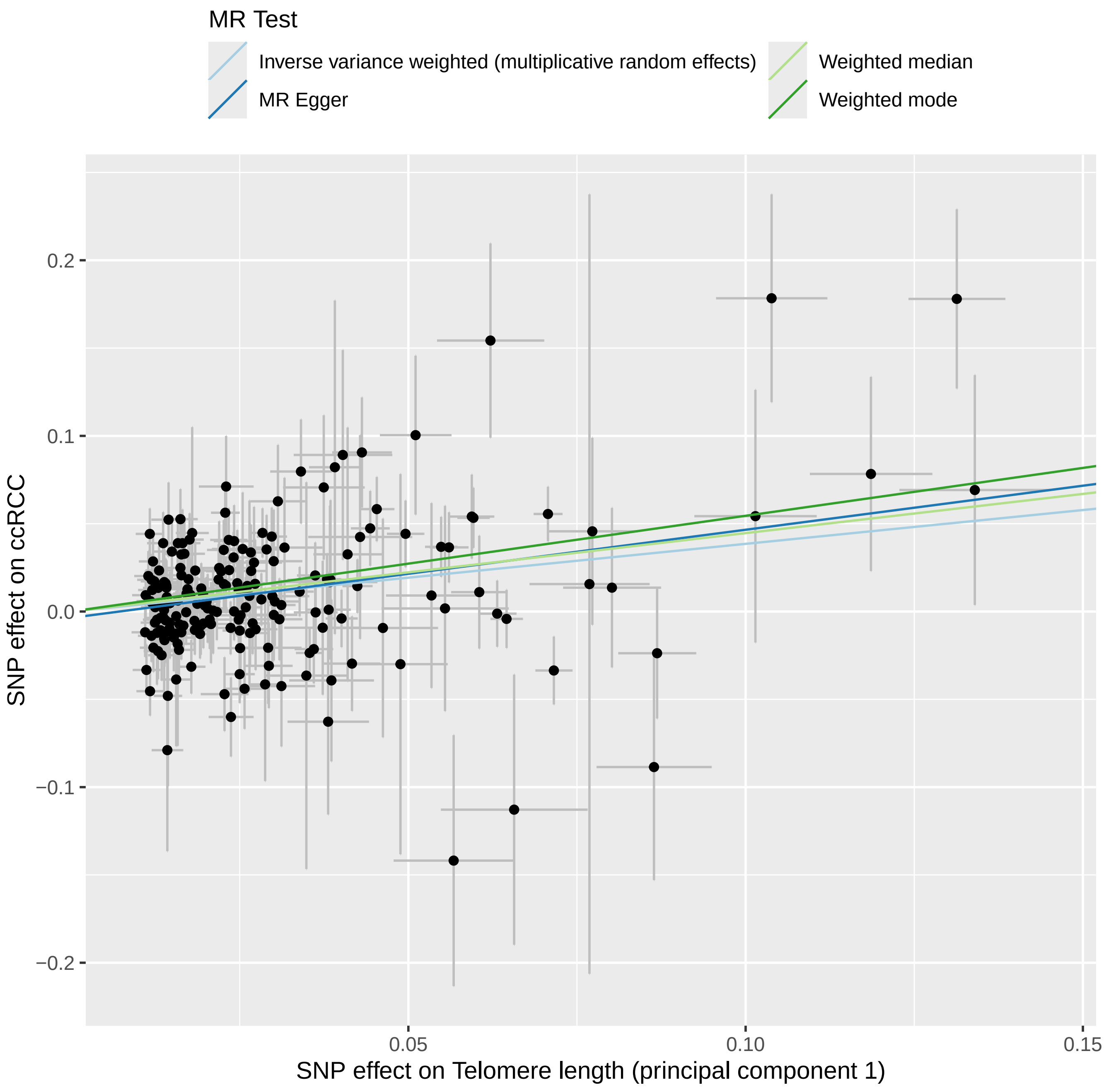
MR Test
Inverse variance weighted (multiplicative random effects)
MR Egger
Weighted median
Weighted mode
0.2
0.1
0.0
−0.1
−0.2
SNP effect on ccRCC
0.05
0.10
0.15
SNP effect on Telomere length (principal component 1)

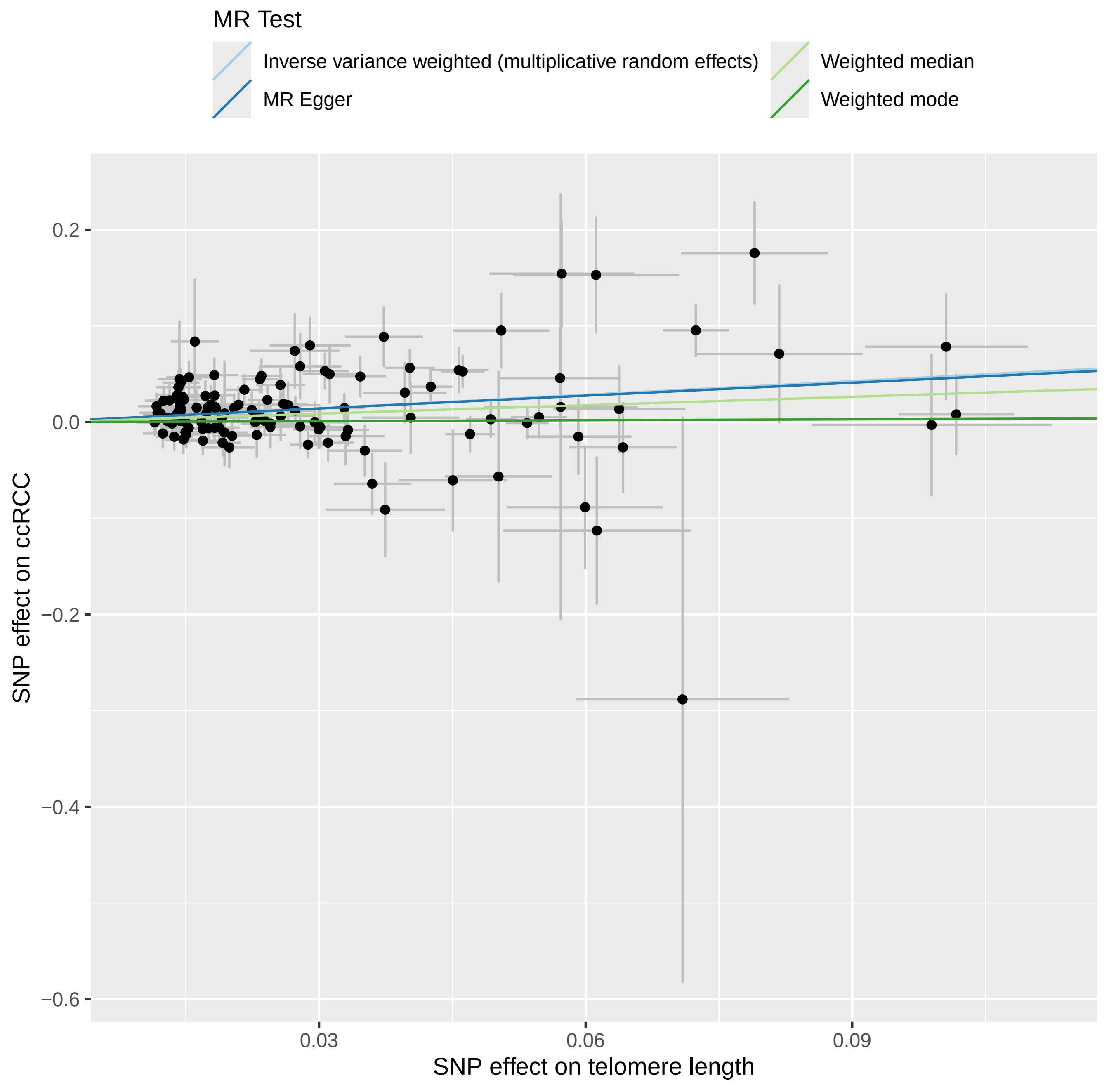

MR Test
Inverse variance weighted (multiplicative random effects)
MR Egger
Weighted median
Weighted mode
0.2
0.0
−0.2
−0.4
−0.6
SNP effect on ccRCC
0.03
0.06
0.09
SNP effect on telomere length

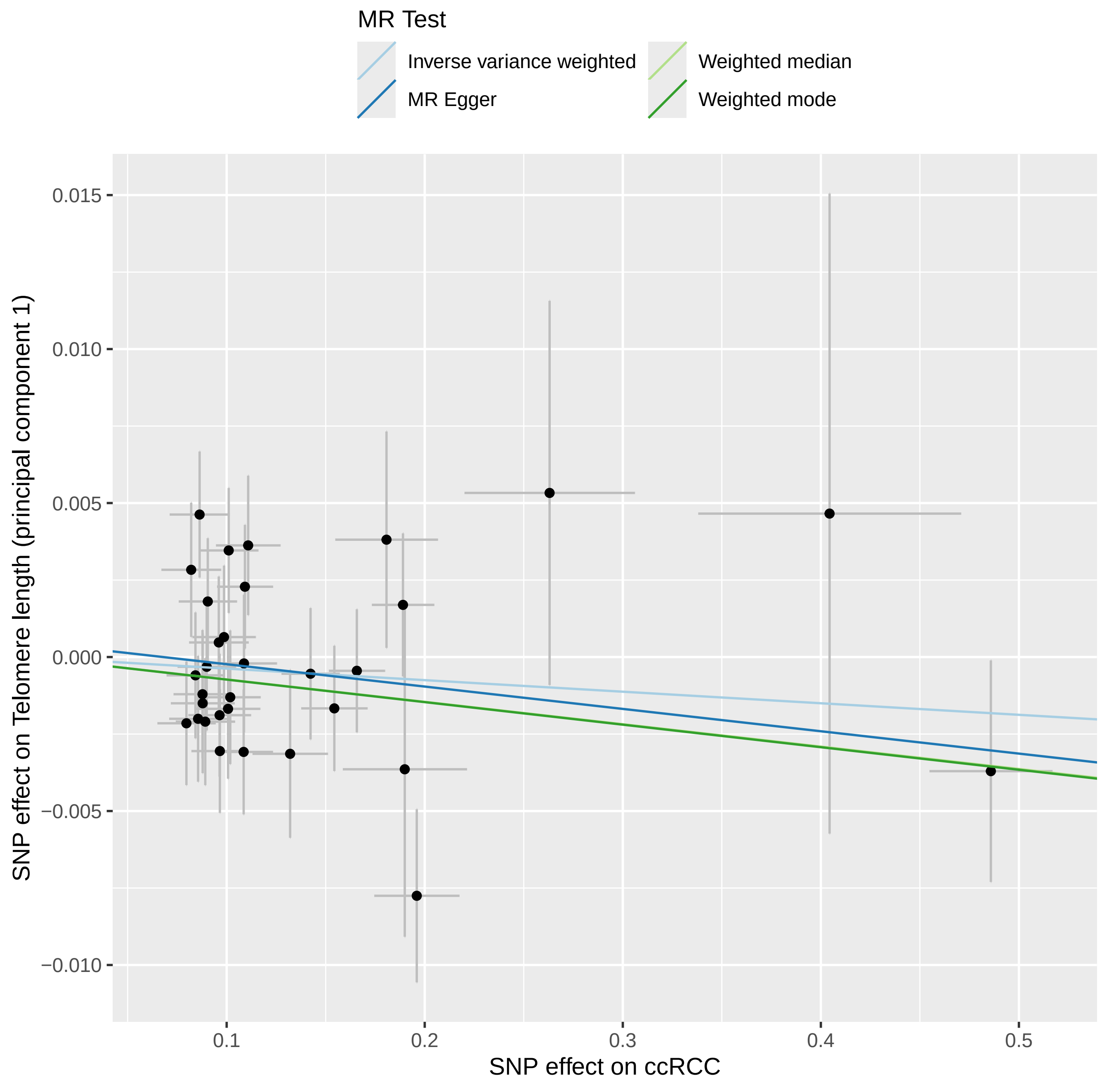
MR Test
Inverse variance weighted
MR Egger
Weighted median
Weighted mode
0.015
0.010
0.005
0.000
−0.005
−0.010
SNP effect on Telomere length (principal component 1)
0.1
0.2
0.3
0.4
0.5
SNP effect on ccRCC

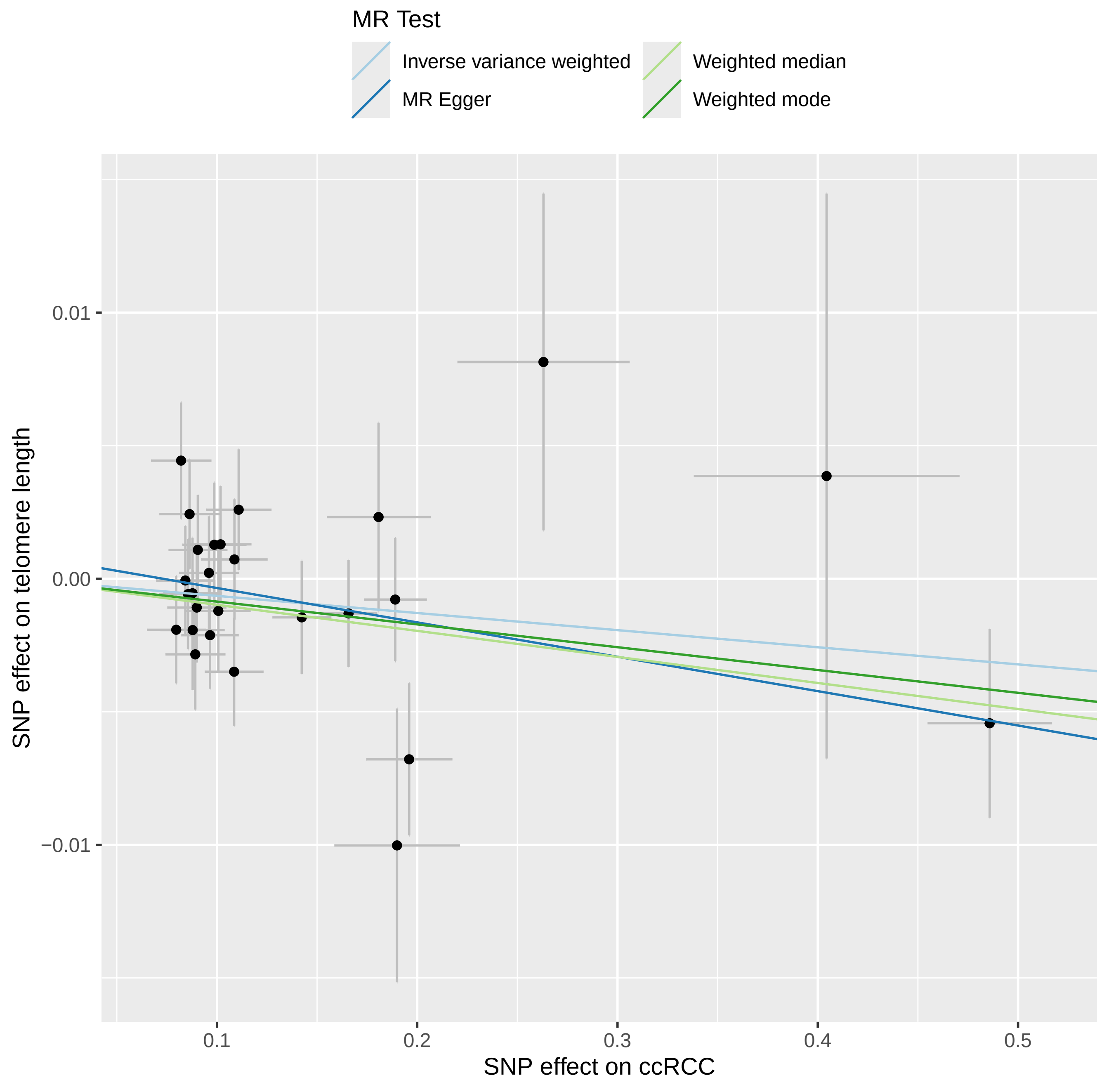
MR Test
Inverse variance weighted
MR Egger
Weighted median
Weighted mode
0.01
0.00
−0.01
0.1
0.2
0.3
0.4
0.5
SNP effect on telomere length
SNP effect on ccRCC
