# Supplementary figures and images for: Telomere Length and Clear Cell Renal Cell Carcinoma: Unraveling Causal Mechanisms Through Integrative Genetic and Single-Cell Transcriptomic Analysis
Source: Mediators Inflamm. 2025 Nov 27;2025:3705788. doi: 10.1155/mi/3705788 (PMC12677994; doi:10.1155/mi/3705788)

All – Inverse variance weighted

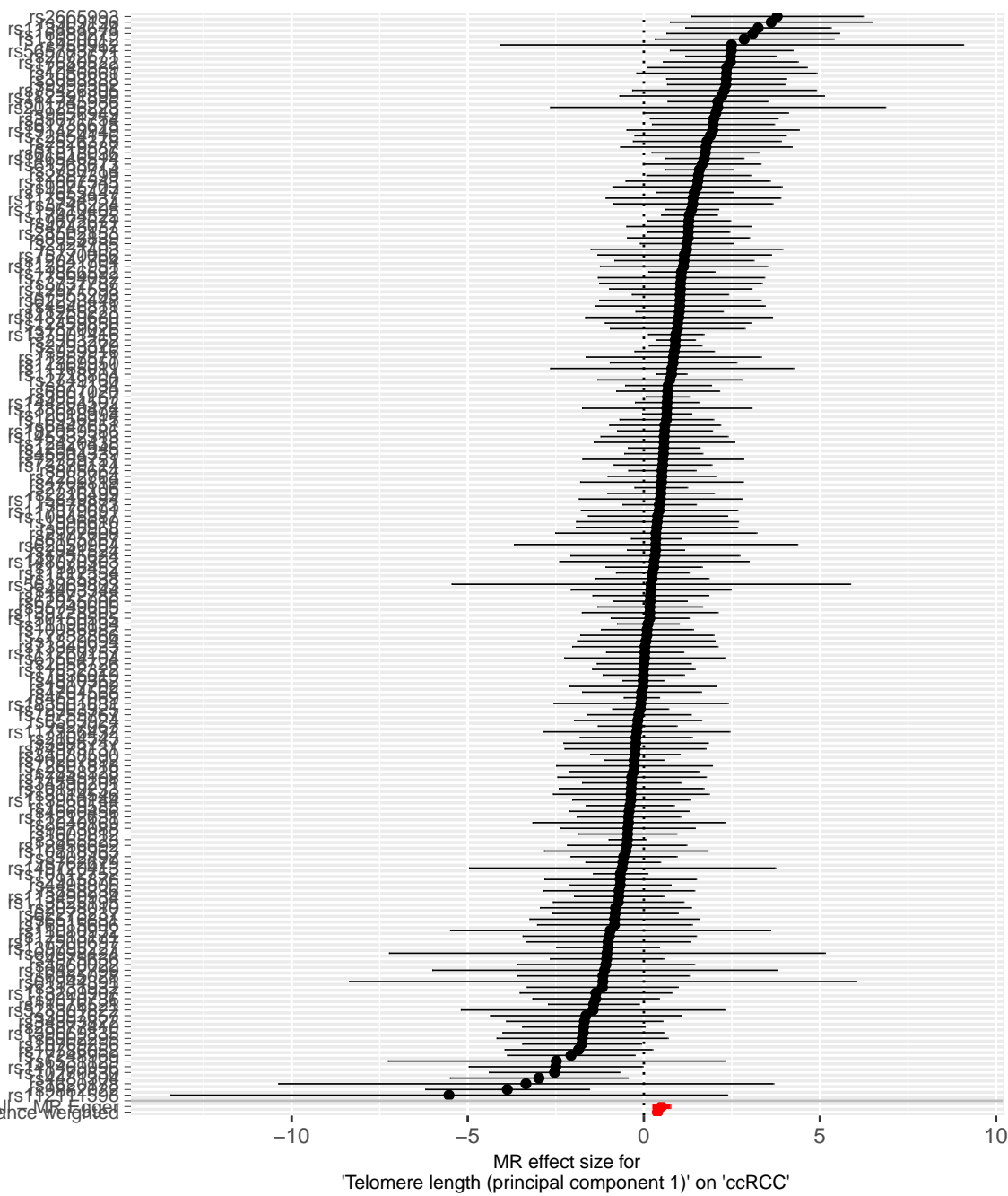

All – Inverse variance weighted

All – MR Egger

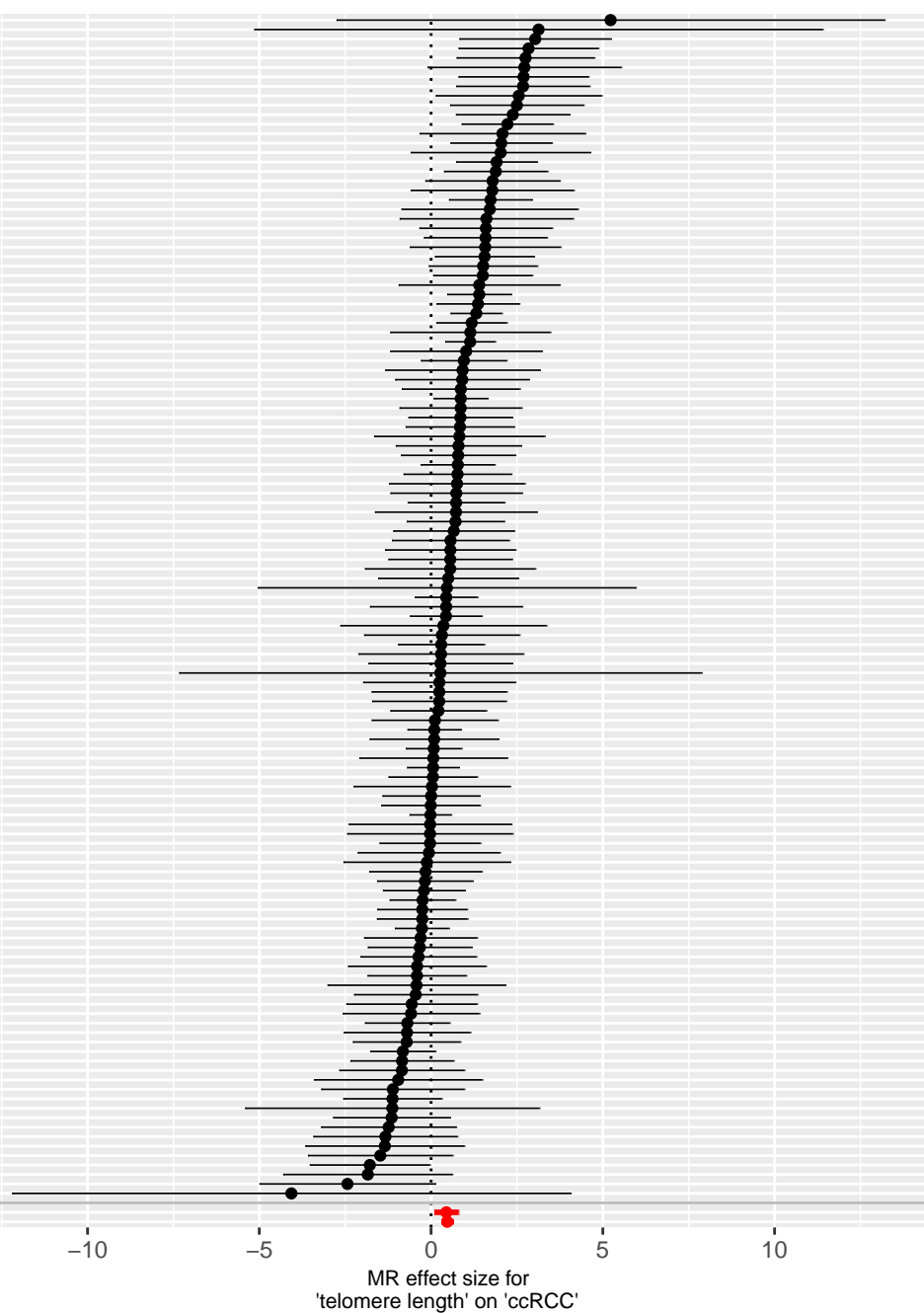

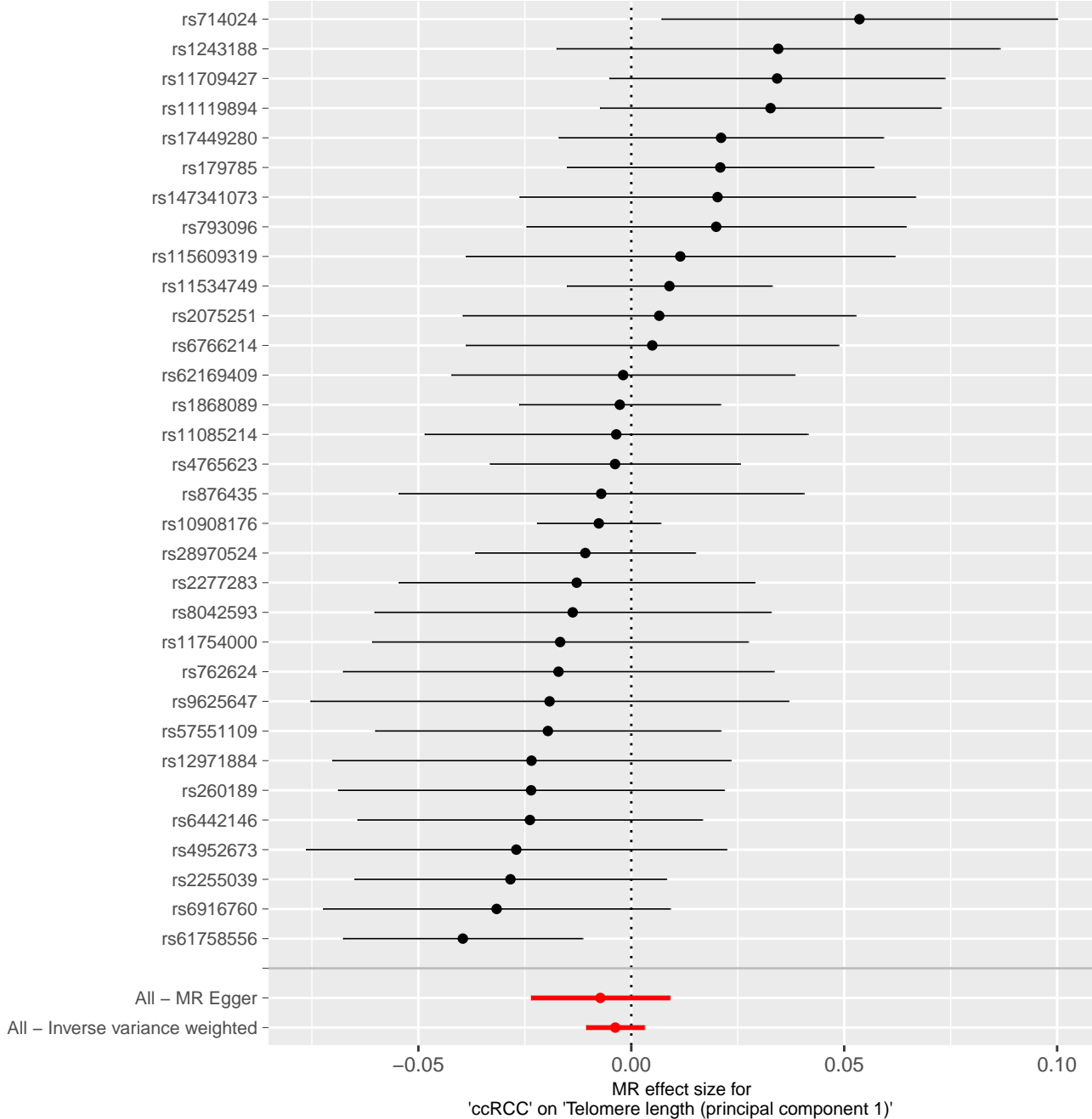

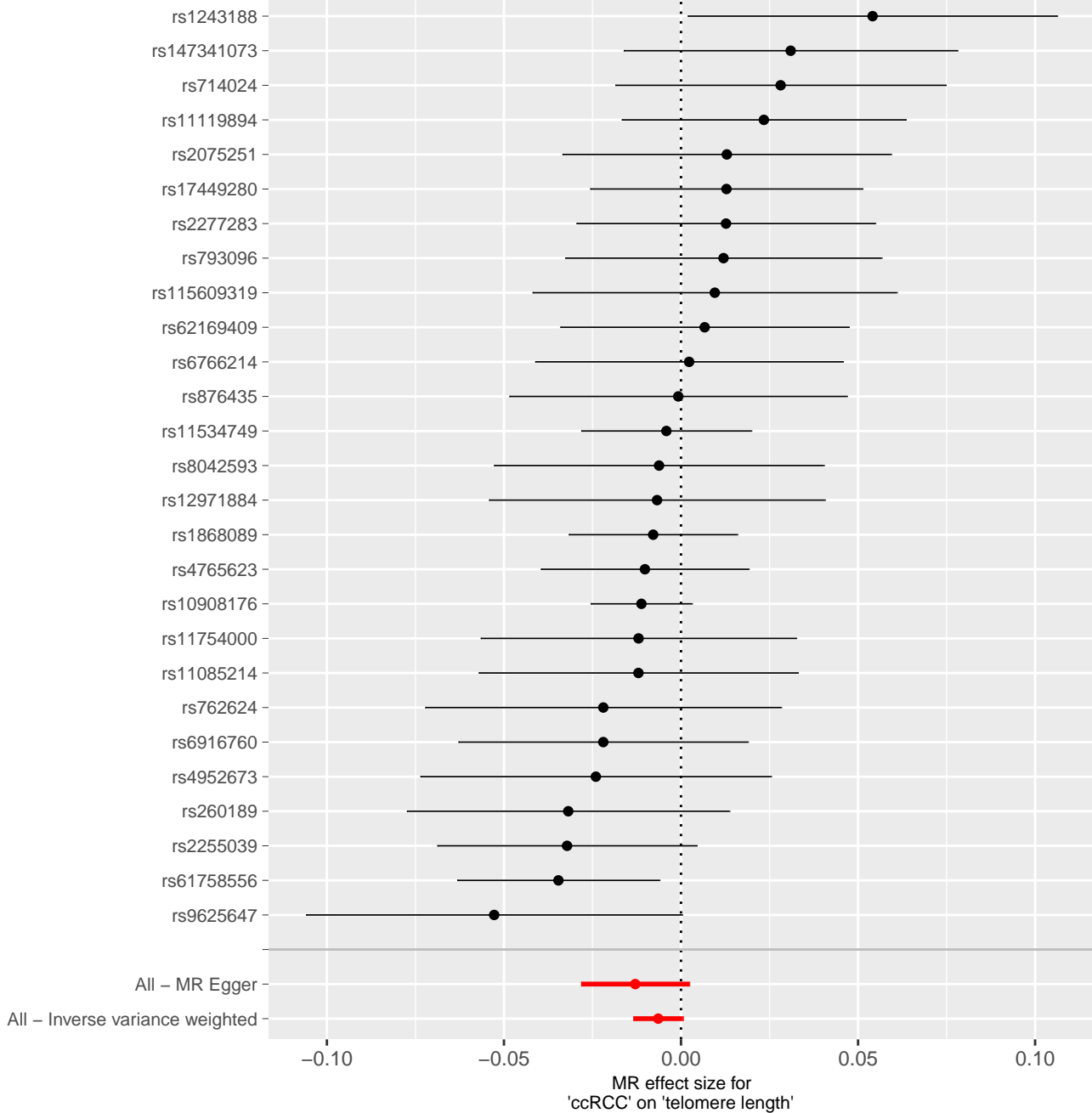

Supplement: Supporting Information 4 — Figure S3. Forest plots. [file 3705788.f4.pdf]

CellAge\_UCell

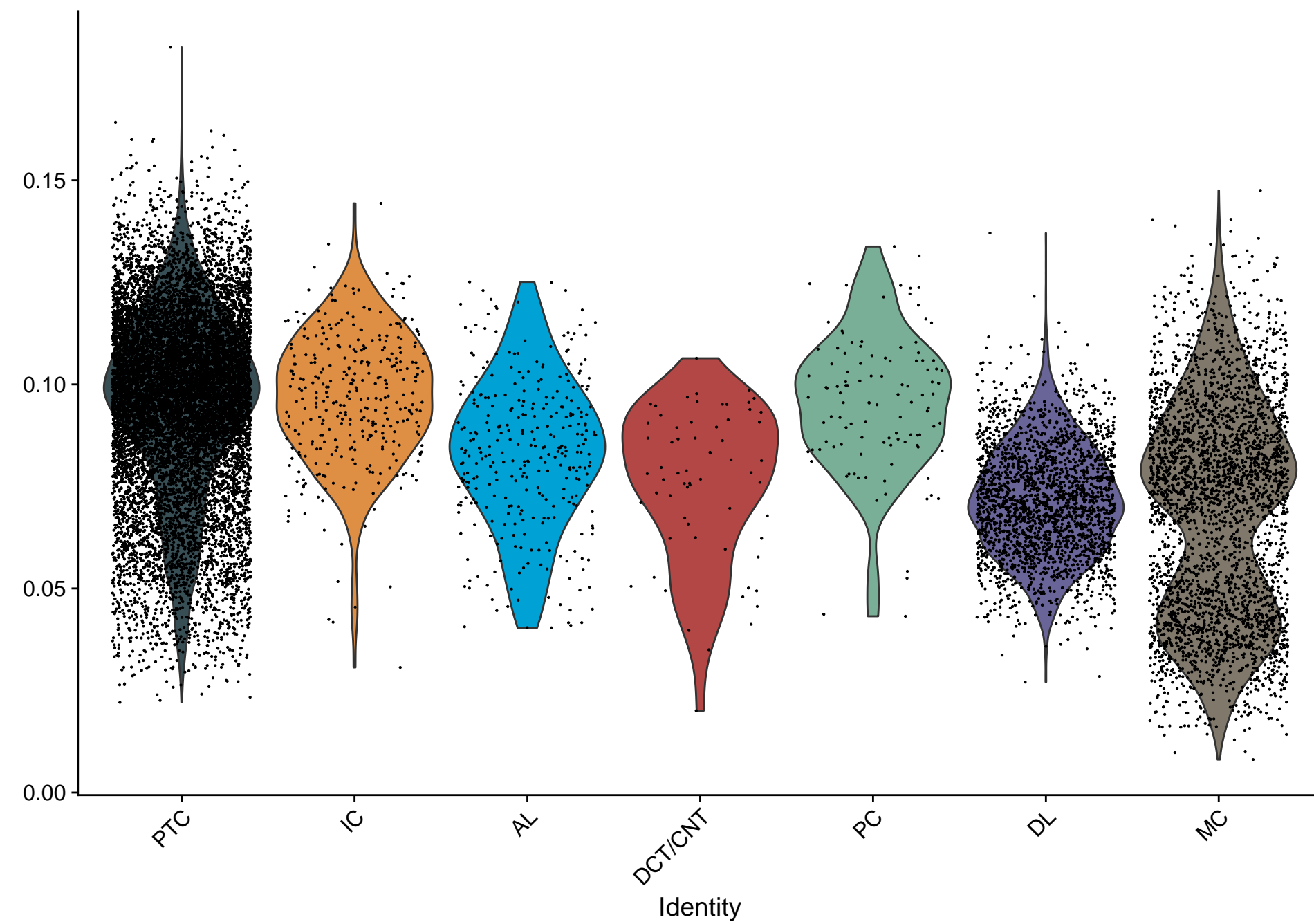

SenMayo\_UCell

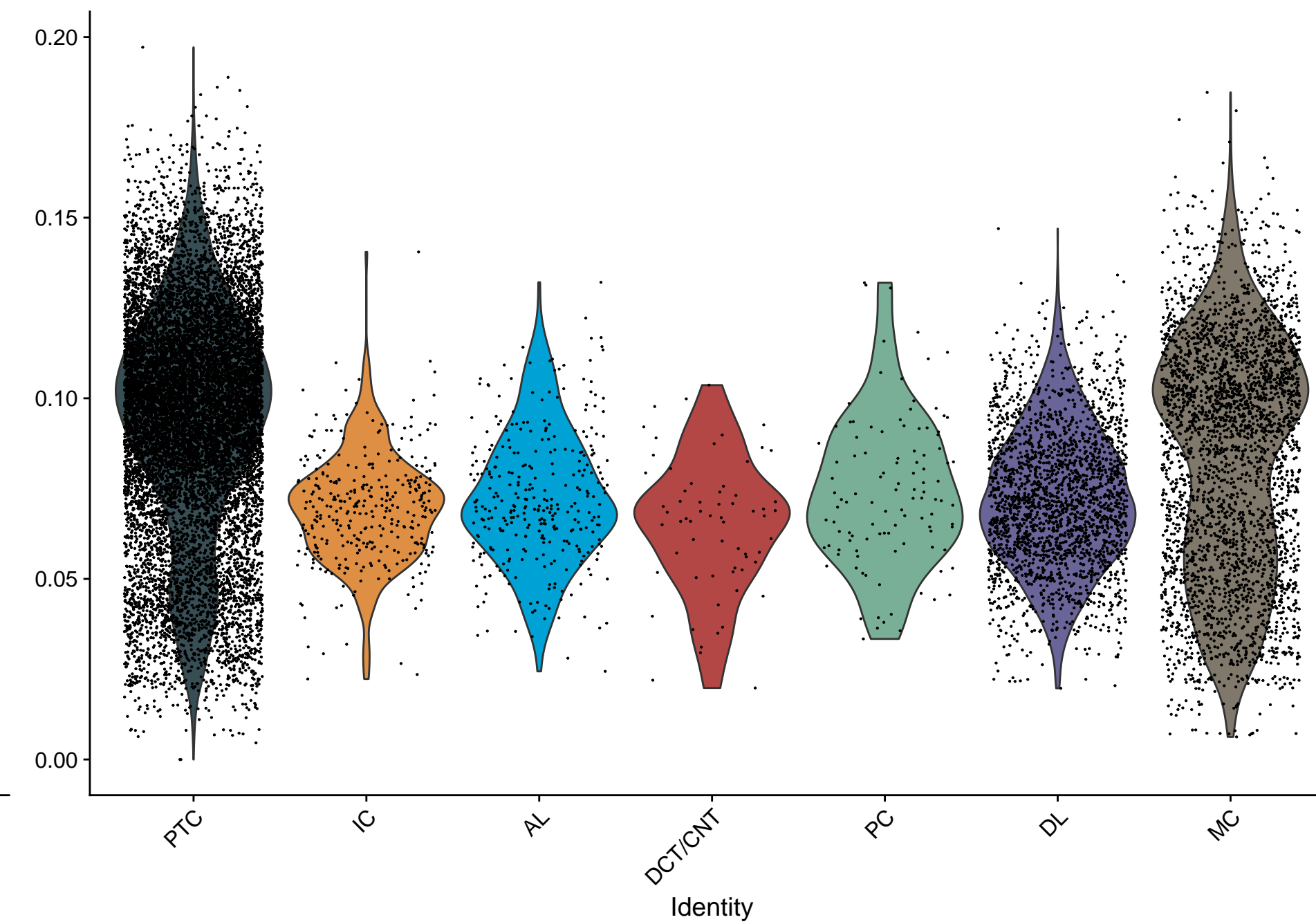

Fridman\_UCell

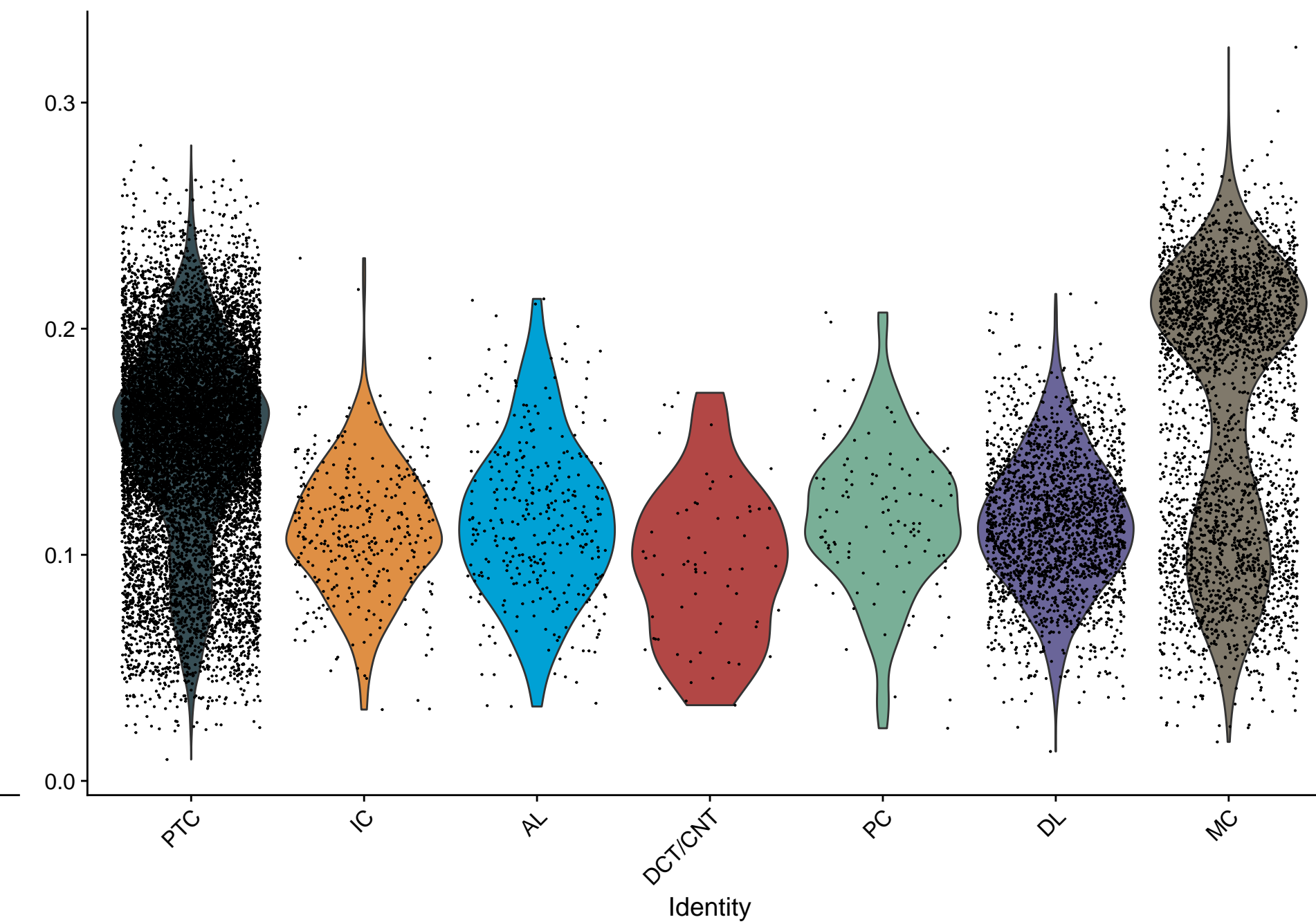

Supplement: Supporting Information 5 — Figure S4. DNA-binding motif analysis and base stacking patterns for NOP10 and NHP2 regulatory regions. [file 3705788.f5.pdf]
